# A novel role for the actin-binding protein drebrin in regulating opiate addiction

**DOI:** 10.1038/s41467-019-12122-8

**Published:** 2019-09-12

**Authors:** Jennifer A. Martin, Craig T. Werner, Swarup Mitra, Ping Zhong, Zi-Jun Wang, Pedro H. Gobira, Andrew. F. Stewart, Jay Zhang, Kyra Erias, Justin N. Siemian, Devin Hagarty, Lauren E. Mueller, Rachael L. Neve, Jun-Xu Li, Ramesh Chandra, Karen C. Dietz, Mary Kay Lobo, Amy M. Gancarz, Zhen Yan, David M. Dietz

**Affiliations:** 1 Department of Pharmacology and Toxicology, Program in Neuroscience, Research Institute on Addictions, The State University of New York at Buffalo, Buffalo, NY 14214 USA; 2 Department of Physiology and Biophysics, The State University of New York at Buffalo, Buffalo, NY 14214 USA; 3 Department of Physics and Chemistry, School of Pharmaceutical Sciences of Ribeirão Preto, University of São Paulo, Ribeirão Preto, São Paulo, Brazil; 4 Department of Psychology, California State University Bakersfield, Bakersfield, CA 93311 USA; 5 Gene Delivery Technology Core, Massachusetts General Hospital, Cambridge, MA 02139 USA; 6 Department of Anatomy and Neurobiology, University of Maryland School of Medicine, Baltimore, MD 21201 USA; 7 Department of Psychology, The State University of New York at Buffalo, Buffalo, NY 14214 USA

**Keywords:** Neuroscience, Reward

## Abstract

Persistent transcriptional and morphological events in the nucleus accumbens (NAc) and other brain reward regions contribute to the long-lasting behavioral adaptations that characterize drug addiction. Opiate exposure reduces the density of dendritic spines on medium spiny neurons of the NAc; however, the underlying transcriptional and cellular events mediating this remain unknown. We show that heroin self-administration negatively regulates the actin-binding protein drebrin in the NAc. Using virus-mediated gene transfer, we show that drebrin overexpression in the NAc is sufficient to decrease drug seeking and increase dendritic spine density, whereas drebrin knockdown potentiates these effects. We demonstrate that drebrin is transcriptionally repressed by the histone modifier HDAC2, which is relieved by pharmacological inhibition of histone deacetylases. Importantly, we demonstrate that heroin-induced adaptations occur only in the D1^+^ subset of medium spiny neurons. These findings establish an essential role for drebrin, and upstream transcriptional regulator HDAC2, in opiate-induced plasticity in the NAc.

## Abstract (web summary)

The underlying transcriptional and cellular events mediating the reduction of dendritic spines on medium spiny neurons of the nucleus accumbens (NAc) remains unknown. Here, authors demonstrate that heroin self-administration negatively regulates the actin-binding protein drebrin in the NAc, which is shown to be transcriptionally repressed by the histone modifier HDAC2, and that overexpression of drebrin is sufficient to decrease drug seeking and increase dendritic spine density

## Introduction

One of the most difficult challenges in the treatment of opiate addiction is the life-long propensity to relapse^1^, contributing to the nearly 50,000 cases of overdose each year^2^. Drug relapse is often triggered by acute re-exposure to the drug, which also occurs in preclinical rodent models of addiction. Opiate-induced relapse is mediated by maladaptive cellular plasticity, including synaptic rewiring, in the nucleus accumbens (NAc) and several other brain regions of the mesolimbic dopamine system. The adaptions to neuronal morphology are also a hallmark of many other psychiatric disorders^3–5^. Synaptic strength, number of synaptic contacts, and glutamate release following abstinence from heroin have been implicated in neuronal plasticity^6,7^. Despite these advances, the precise molecular mechanisms responsible for the cellular and behavioral plasticity remain elusive.

The enduring adaptations observed in response to drugs of abuse, particularly in medium spiny neurons (MSNs) in the striatum, have been attributed to alterations in gene transcription regulated through epigenetic-mediated mechanisms^8^. Opiates^9–11^ and psychostimulants^12,13^ induce disparate molecular, transcriptional, and functional changes in the two MSN subtypes, those expressing dopamine receptor 1 (D1) or dopamine receptor 2 (D2), which project to midbrain and pallidal regions or pallidal regions only, respectively^14–17^.

The structural changes to neuronal dendrites, including those following exposure to drugs of abuse, are driven by actin dynamics^18–20^, a pathophysiological pathway common to neuropsychiatric disorders. Actin cycling is tightly controlled by robust transcription and epigenetic regulation of genes encoding cytoskeletal proteins^21,22^. One such protein is drebrin, which modulates both dendritic spine plasticity and synaptic function and is dysregulated in certain disease states^23,24^. However, the defining transcriptional and cell-type-specific regulators of actin dynamics that influence dendritic spine plasticity and actin homeostasis in MSNs under the influence of drugs of abuse remain largely unknown.

Gene expression is regulated, in part, through chromatin remodeling. The regulation of the chromatin state by histone acetylation drives drug-induced alterations of gene expression contributing to maladaptive behaviors^25^. Histone deacetylases (HDACs) are enzymes that tightly regulate this process, and HDAC inhibitors have gained momentum as potential therapeutics for neuropsychiatric diseases, such as depression^26^ and anxiety disorders^27^. However, the role of HDACs in mediating opioid-induced cellular plasticity and the therapeutic potential of HDAC inhibition in heroin abuse remain understudied.

Here, we demonstrate the epigenetic (HDAC2) and cell-type-specific (D1/D2) regulation of drebrin expression following opioid exposure, ultimately resulting in drug-induced changes in actin stability. Our results demonstrate an essential role of drebrin in heroin-induced behavioral and structural plasticity in the NAc. The studies presented here are among the first to implicate transcriptional regulation of structural plasticity, in a cell-type-specific manner, mediating susceptibility to relapse, thus providing a pathway for intervention across several domains of plasticity.

## Results

### Opiates alter actin dynamics in the NAc

**Fig. 1:**
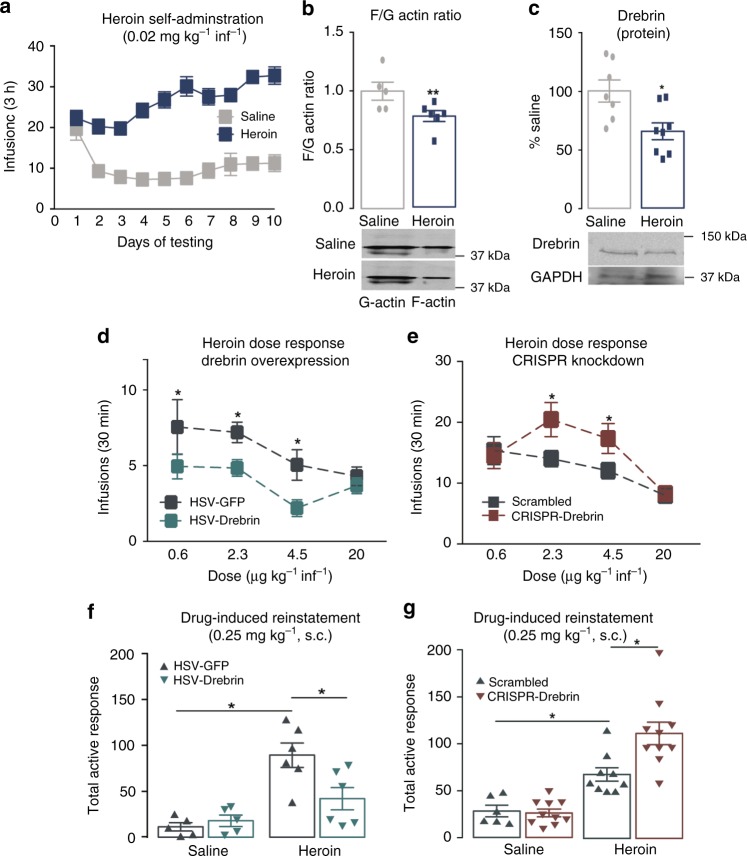
Drebrin mediates behavioral plasticity in the NAc. **a** Mean numbers of infusions of saline (gray) or heroin (blue) self-administered over 10 days (two-way repeated-measures ANOVA: drug effect, F_1,41_ = 76.74, *P* < 0.001; day effect, F_9,369_ = 7.899, *P* < 0.001; interaction, F_9,369_ = 8.882, *P* < 0.001, *n* *=* 19–24 per group). Actin cycling (Student’s *t*-test: *t*_9_ = 2.632, *P* = 0.027, *n* *=* 6–7 per group) (**b**) and drebri*n* protein levels (Student’s *t*-test*: t*_13_ = 2.953, *P* *=* 0.011, *n* *=* 7–8 per group) (**c**) in the NAc following heroin SA. Within-session heroin SA dose response (μg kg^−1^ infusion^−1^ [inf]) following intra-NAc injections of HSV-drebrin or HSV-GFP (two-way ANOVA: dose effect, F_3,72_ = 4.314, *P* < 0.001; virus effect, F_1,72_ = 10.68, *P* < 0.01; interaction, F_3,71_ = 0.56, *P* *>* 0.05, *n* *=* 10–11 per group) (**d**) and HSV-CRISPR-Cas9 drebrin (CRISPR-drebrin) or HSV-scrambled (two-way ANOVA: dose effect, F_3,60_ = 8.796, *P* < 0.001; virus effect, F_1,60_ = 4.467, *P* < 0.05; interaction, F_3,60_ = 1.864, *P* *>* 0.05, *n* *=* 9–8 per group) (**e**). Total active responses during drug-induced reinstatement following virus-mediated overexpression of drebrin (two-way ANOVA: drug effect, F_1,18_ = 23.68, *P* < 0.001; virus effect, F_1,18_ = 3.782, *P* *=* 0.068; interaction, F_1,18_ = 6.63, *P* *=* 0.019; saline-GF*P* vs heroin-GFP, *P* *=* 0.003; heroin-GFP vs heroin-drebrin, *P* *=* 0.017; saline-GFP vs heroin-drebrin, *P* *=* 0.201; *n* *=* 5–6 per group) (**f**) or CRISPR-drebrin (two-way ANOVA: drug effect, F_1,31_ = 53.71, *P* < 0.001; virus effect, F_1,31_ = 5.937, *P* < 0.05; interaction, F_1,31_ = 7.17, *P* *=* 0.01, saline-scrambled vs heroin-scrambled, *P* = 0.02; heroin-scrambled vs heroin-CRISPR, *P* = 0.003; *n* *=* 6–10 per group) (**g**) in the NAc. Data are expressed as means ± SEMs. *P < 0.05, **P < 0.01

To investigate alterations in actin dynamics in response to drugs of abuse, we trained rats to self-administer (SA) heroin (Fig. 1a), which decreases the density of dendritic spines on MSNs in the NAc shell (Supplementary Fig. 1a). We found altered actin cycling in NAc MSNs, observed as a decrease in the filamentous-to-globular actin ratio (F/G ratio) (Fig. 1b), which was driven by a decrease in F-actin pools in the NAc (Supplementary Fig. 1b, c). We next sought to determine the critical mediators of this altered actin stability.

As reduced expression of the actin-binding protein drebrin, which bundles actin filaments to maintain synaptic integrity, has been implicated in the pathogenesis of conditions marked by dendritic spine loss (i.e., Alzheimer’s, schizophrenia)^28,29^, we assessed drebrin expression following opiate exposure. Following heroin SA, mRNA and protein levels of drebrin were decreased in the NAc (Fig. 1c and Supplementary Fig. 2), but not in the caudate putamen of the striatum (Supplementary Fig. 3a). In contrast, drebrin expression was not altered following sucrose SA (Supplementary Fig. 3b), suggesting the changes in expression may be specific to heroin exposure.

### Drebrin bidirectionally regulates heroin-induced plasticity

To determine a physiological role of drebrin in the regulation of addiction-like behaviors, we utilized virus-mediated gene transfer via herpes simplex viruses (HSVs) to overexpress drebrin or green fluorescent protein (GFP) and CRISPR-Cas9 to knockdown drebrin. We first turned to a within-session dose-response paradigm, which allowed us to measure enhanced or decreased sensitivity (e.g., horizontal shifts) or can be predictive of drug vulnerability (i.e., a vertical shifts), across several doses of drug^30^. Specifically, vertical changes in drug reward represent an allostatic change, which has previously been demonstrates in animals based on their intrinsic vulnerability^31^. HSV-mediated overexpression of drebrin (Supplementary Fig. 4a, b) resulted in a vertical downward shift in heroin SA (Fig. 1d), whereas potentiation of the opiate-induced repression of drebrin by HSV-CRISPR-,drebrin (Supplementary Fig. 4c–e) resulted in an upward shift in SA (Fig. 1e), demonstrating a weakening and heightening of drug vulnerability, respectively^31,32^. Given the effects of drebrin expression in a model predictive of addiction vulnerability, we next sought to determine to role of drebrin in relapse-like behaviors. Drebrin overexpression in the NAc was sufficient to attenuate heroin-primed reinstatement (Fig. 1f), which was not due to an overall suppression of locomotor and operant behaviors (Supplementary Fig. 5a, b), whereas HSV-CRISPR-drebrin exacerbated drug-seeking behavior (Fig. 1g). These effects were specific for heroin-related relapse-like behaviors, as sucrose reinstatement was not altered by viral manipulation of drebrin expression (Supplementary Fig. 6a, b).

### HDAC2-mediated regulation of drebrin

**Fig. 2:**
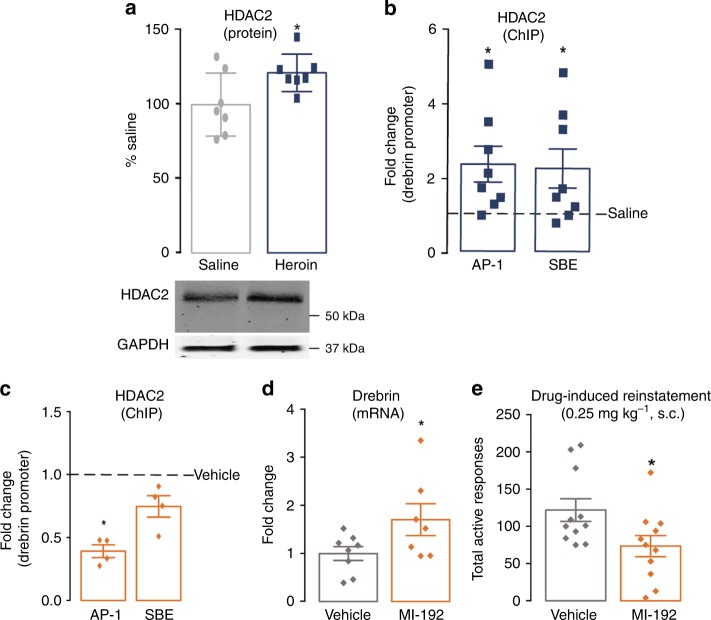
Epigenetic regulation of drebrin expression following heroin SA. HDAC2 protein expression (Student’s t-test: *t*_12_ = 2.279, *P* < 0.05, *n* *=* 7 per group) (**a**) and binding along AP-1 (Student’s *t*-test: *t*_14_ = 2.435, *P* < 0.05, *n* *=* 8 per group) and SBE (Student’s t-test: *t*_14_ = 2.17, *P* < 0.05, *n* *=* 8 per group) sites along the drebrin promoter (**b**) following 10 days of heroin SA. HDAC2 binding along AP-1 (Student’s *t*-test: *t*_6_ = 3.383, *P* < 0.05, *n* *=* 4 per group) and SBE (Student’s *t*-test: t_6_ = 31.313, *P* *=* 0.23, *n* *=* 4 per group) sites on the drebrin promoter (**c**), drebrin mRNA levels (Student’s t-test: *t*_13_ = 2.056, *P* < 0.05, *n* *=* 7–8 per group) (**d**), and total active responses (Student’s *t*-test: *t*_20_ = 2.329, *P* < 0.05, *n* *=* 11 per group) (**e**) following HDAC2 inhibition via MI-192 microinjections. Data are expressed as means ± SEMs. *P < 0.05

To determine the mechanism by which opiates downregulate drebrin expression in the NAc, we first examined the acetylation of the drebrin promoter, as histone acetylation is known to positively regulate the transcription of genes associated with synaptic plasticity^33^. We found that repeated morphine exposure resulted in decreased acetylation on the drebrin promoter (Supplementary Fig. 7a), which was concomitant with a decrease in drebrin expression (Supplementary Fig. 7b, c). Returning to the more translatable model of addiction-like behaviors, we sought to understand the precise mechanism by which opiate SA decreased the acetylation and expression of drebrin. Therefore, we investigated a stimulus-induced transcriptional repressor, HDAC2^34^, as it has been highly implicated in mediating neuronal and synaptic plasticity. HDAC2 expression was upregulated in the NAc after heroin exposure (Fig. 2a) and also had increased binding along AP-1 and Smad binding element (SBE) promoter sites (Fig. 2b), which are critical sites for drug-induced transcriptional plasticity^32,35^. To determine a direct role of HDAC2-mediated drebrin regulation and behavioral responses, the HDAC inhibitor MI-192^36^, which inhibits HDAC2/3 with higher selectivity for HDAC2 (IC_50_ of 16 and 30 nm, respectively), was bilaterally injected directly into the NAc. Intra-NAc inhibition of HDAC decreased HDAC2 binding at AP-1 and SBE sites on the drebrin promoter (Fig. 2c) and increased drebrin mRNA levels (Fig. 2d). Notably, MI-192 in the NAc decreased drug-induced reinstatement (Fig. 2e). Together, these data suggest a transcriptional and epigenetic mechanism by which HDAC2 regulates drebrin expression and behavioral responses to heroin thus revealing a potential therapeutic target to reduce drug-induced relapse-like behaviors.

### Drebrin in the NAc does not alter pain-related behaviors

Opiates are commonly used for their therapeutic and analgesic properties, resulting in drug tolerance with prolonged used^37^. As the NAc is thought to integrate emotional and sensory information^38,39^, we sought to elucidate the role of drebrin in the NAc in opiate-induced pain-related behaviors and tolerance. To this end, we utilized a place escape avoidance paradigm to assess affective (emotional^40^) pain by pairing aversive stimuli with an innately preferred chamber in addition to von Frey testing to assess physical (mechanical) pain. Neither drebrin overexpression nor knockdown altered affective (Supplementary Fig. 6c, d) or physical (Supplementary Fig. 6e, f) pain. Furthermore, manipulation of drebrin expression in the NAc did not alter the induction of tolerance to the analgesic effects of opiates (Supplementary Fig. 6e, f), suggesting that drebrin in the NAc is not a critical regulator of the analgesic properties of opiates and is specific for addiction-like behaviors.

### Drebrin rescues heroin-induced synaptic deficits in the NAc

**Fig. 3:**
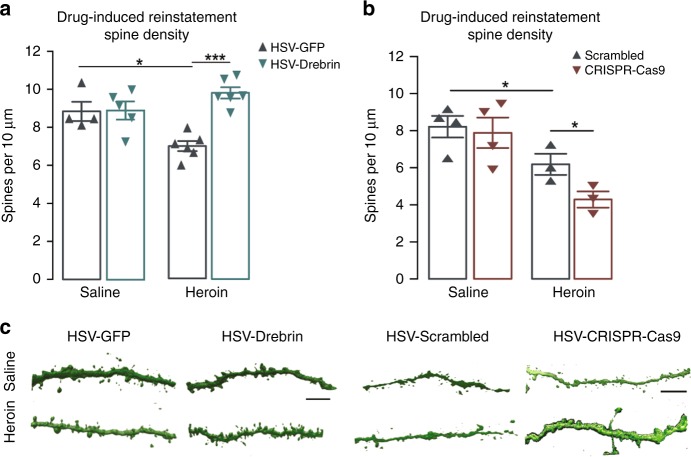
Drebrin in the NAc regulates opiate-induced structural plasticity. **a** Spine densities on MSNs in the NAc following intra-NAc HSV-drebrin expression (two-way ANOVA: drug effect, F_2,15_ = 0.482, *P* < 0.05; virus effect, F_1,15_ = 13.71, *P* *>* 0.05; interaction, F_2,15_ = 5.91, *P* < 0.01, saline-GFP vs heroin-GFP, *P* *=* 0.008; heroin-GFP vs heroin-drebrin, *P* *=* 0.0002; saline-GFP vs heroin-drebrin, *P* *=* 0.125; *n* = 5–6 per group). **b** Spine de*n*sities on MSNs in the NAc following intra-NAc CRISPR-Cas9 drebrin (two-way ANOVA: drug effect, F_1,10_ = 20.92, *P* < 0.001; virus effect, F_1,10_ = 3.858, *P* *>* 0.05; interaction, F_1,10_ = 2.138, *P* *>* 0.05; saline-scrambled vs heroin-scrambled, *P* = 0.05; heroin-scrambled vs heroin-CRISPR, *P* = 0.04; *n* = 3–4 per group). **c** Representative images of virus-infected neurons; scale bars, 5 μm. Data are expressed as means ± SEMs. *P < 0.05, ***P < 0.001

As drebrin is an important mediator of structural plasticity, we aimed to determine its role in heroin-induced spine plasticity. The decrease in spine density on MSNs following heroin SA was reversed by HSV-mediated drebrin overexpression (Fig. 3a, c), particularly through the restoration of thin spines (HSV-GFP, 0.69 spines per 10 μM ± 0.848; HSV-drebrin, 2.63 spines per 10 μM ± 1.77; Student’s t-test: *t*_16_ = 2.934, *P* < 0.05). Conversely, HSV-CRISPR-drebrin potentiated heroin-induced decreases in spine density (Fig. 3b, c), an effect mediated via thin spines (HSV-scrambled, 3.15 spines per 10 μM ± 0.18; HSV-CRISPR-Cas9, 2.13 spines per 10μM ± 0.17; Student’s t-test: *t*_10_ = 2.238, *P* < 0.05). Dendritic spine density was negatively correlated with total active responses during reinstatement with both drebrin overexpression and drebrin knockdown (Supplementary Fig. 8a, b), suggesting that the modulation of spines mediates reinstatement behaviors.

**Fig. 4:**
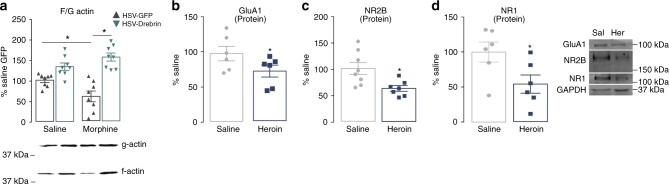
Drebrin restores opiate-induced deficits in synaptic plasticity. **a** Intra-NAc expression of HSV-drebrin rescues morphine-induced changes in F/G-actin ratios (two-way ANOVA: drug effect, F_1,28_ = 0.979, *P* > 0.05; virus effect, F_1,28_ = 45.14, *P* < 0.01; interaction, F_1,28_ = 10.62, *P* < 0.01; saline-GFP vs morphine-GFP, *P* = 0.005; morphine-GFP vs morphine-drebrin, *P* < 0.0001; saline-GFP vs morphine-drebrin, *P* = 0.0004; saline-GFP vs saline-drebrin, *P* = 0.02; *n* = 8 per group). Surface protein expressio*n* of AMPA receptor subunit GluA1 (Student’s t-test: *t*_10_ = 1.949, *P* < 0.05, *n* = 6 per group) (**b**) and NMDA receptor subunits NR2B (Student’s t-test: *t*_13_ = 2.856, *P* < 0.05, *n* = 7–8 per group) (**c**) and NR1 (S*t*udent’s t-test: *t*_10_ = 2.405, *P* < 0.05, *n* = 6 per group) (**d**) in the NAc following heroin SA. Data are expressed as means ± SEMs. *P < 0.05

Actin cycling governs dendritic spine formation, structure, and maintenance^41^. We therefore examined whether drebrin overexpression was sufficient to normalize opiate-induced decreases in actin stability. Using repeated morphine exposure, which allowed for a temporal regulation of both viral expression and opiate exposure, we showed that F/G-actin ratios were restored by HSV-drebrin (Fig. 4a), which occurred by reversing the opiate-induced decrease in F-actin pools (Supplementary Fig. 9a, b). These actin dynamic-driven alterations in spine density likely altered postsynaptic glutamatergic signaling, which is in part governed by cytoskeletal proteins^42^. Consistent with the loss of dendritic spines induced by heroin, we observed decreases in the surface expression of AMPA and NMDA receptor subunits after SA (Fig. 4b–d). Electrophysiological analyses confirmed that heroin SA decreased both AMPA (Supplementary Fig. 10c) and NMDA (Supplementary Fig. 10d) receptor excitatory postsynaptic currents but did not alter AMPA/NMDA current ratios (Supplementary Fig. 10a; representative traces Supplementary Fig. 10b). Importantly, virus-mediated drebrin expression, which rescued heroin-induced spine density loss, also restored AMPA and NMDA receptor currents (Supplementary Fig. 10c, d).

### Opiate-induced plasticity in D1-MSNs

**Fig. 5:**
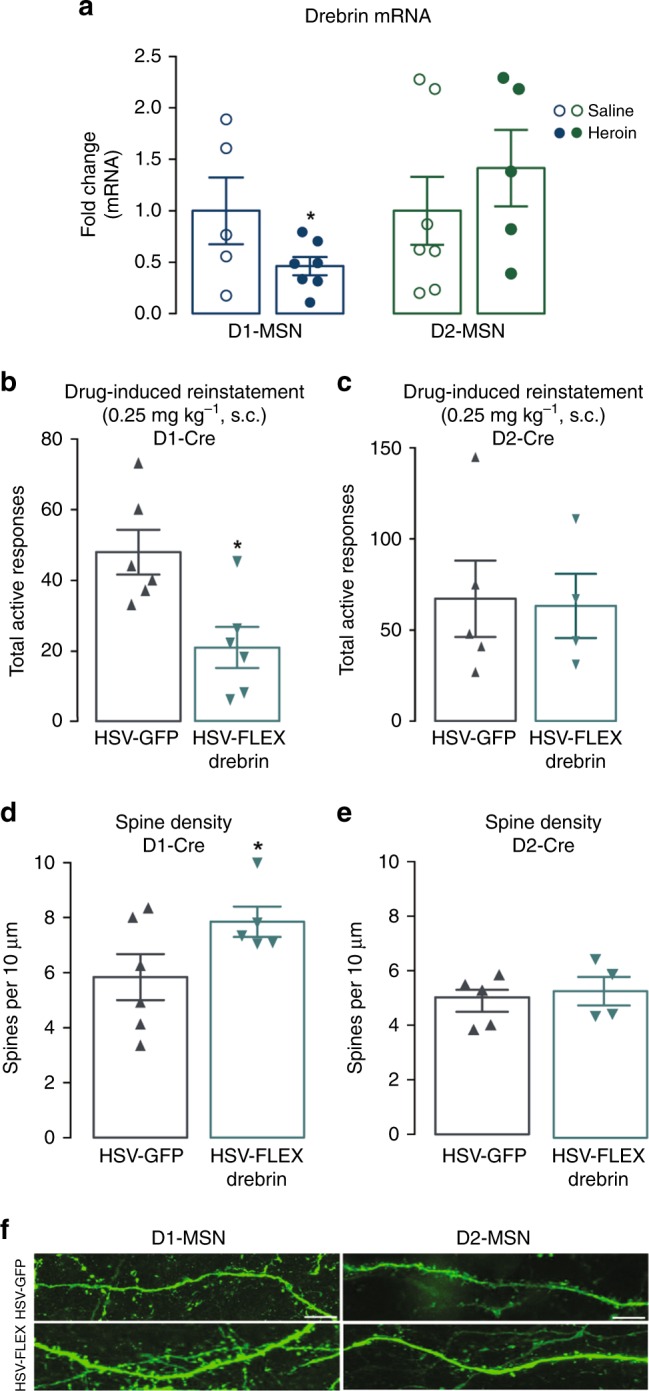
Cell-type-specific expression of drebrin alters behavioral and structural plasticity. **a** Drebrin mRNA expression in D1-expressing (Student’s t-test: *t*_10_ = 1.864, *P* < 0.05, *n* = 5–7 per group) and D2-expressing (Student’s *t*-test: *t*_10_ = 0.82, *P* > 0.05, *n* = 5–7 per group) MSNs following heroin SA. Total active responses following cell-type-specific overexpression of drebrin in D1-expressing (Student’s t-test: *t*_10_ = 3.153, *P* < 0.05, *n* = 6 per group) (**b**) or D2-expressing (Student’s *t*-test: *t*_7_ = 0.1394, *P* > 0.05, *n* = 4–5 per group) (**c**) MSNs. Dendritic spine densities following **c**ell-type-specific overexpression of drebrin in D1-expressing (Student’s *t*-test: *t*_9_ = 1.914, *P* < 0.05, *n* = 5–6 per group) (**d**) or D2-expressing (Student’s *t*-test: *t*_7 _= 0.537, *P* > 0.05, *n* = 4–5 per group) (**e**) MSNs. (**f**) Representative images of virus-infected neurons; scal**e** bars, 5 μm. Data are expressed as means ± SEMs. *P < 0.05

As D1 and D2 MSNs have selective and often opposing roles in responses to drugs of abuse^16^, we next examined whether the expression of drebrin in the NAc and drebrin-mediated plasticity following exposure to opiates was cell-type specific. Using validated transgenic RiboTag technology, we found that following heroin SA (Fig. 5a) in D1- and D2-cre transgenic rats^43^ (Supplementary Fig. 11) or repeated administration of morphine (Supplementary Fig. 12) in D1- and D2-ribotag mice, drebrin expression decreased exclusively in D1-type MSNs in the NAc. These data also demonstrate that cell-type-specific regulation of drebrin is conserved across species (mice, rats), opiate drug (morphine, heroin), and paradigm of drug administration (nonvolitional, volitional). In agreement with our whole-cell findings demonstrating that HDAC2 regulates drebrin expression, we found that HDAC2 was upregulated in D1 but not D2 MSNs (Supplementary Fig. 13) following heroin SA. To determine if the cell-type-specific decreases in drebrin expression extend to heroin-induced behavioral and structural plasticity, we utilized a Cre-inducible viral vector to overexpress drebrin in Cre-expressing D1 or D2 MSNs. Drebrin overexpression in D1 but not D2 MSNs attenuated drug-induced reinstatement (Fig. 5b, c) and heroin-induced decreases in dendritic spine density (Fig. 5d–f), with a restoration of thin spines (HSV-GFP, 4.033 spines per 10μM ± 0.36; HSV-LS1L-drebrin, 5.34 spines per 10 μM ± 0.51; Student’s t-test: *t*_9_ = 2.152, *P* < 0.05). These data are among the first to highlight a molecular basis for cell-type-specific plasticity following heroin abuse.

## Discussion

We demonstrated that the actin-binding protein drebrin is downregulated in the NAc following heroin administration. Furthermore, we found that drebrin is transcriptionally repressed by the histone modifier HDAC2, an effect that was reversed by pharmacological inhibition of HDACs. Importantly, we demonstrated that the downregulation of drebrin occurs exclusively in D1 MSNs and that restoration of drebrin levels in these cells relieves opiate-induced structural changes and diminishes drug-induced craving (Supplementary Fig. 14). Taken together, the results from this study provide the first evidence of a detailed cellular mechanism of relapse to opiates in a cell-type- and circuit-specific manner.

Alterations in synaptic connectivity are a hallmark of many psychiatric disease states, including opioid use disorder^19,42,44^. The excitatory synaptic connections on dendritic spines, including those in the NAc, are modulated by actin cycling^45^. In this study, we found that heroin SA disrupts this finely tuned process, resulting in changes to dendritic spine structure and function in the NAc shell. The observed decrease in glutamatergic tone, through both AMPA and NMDA receptor surface expression and currents, is consistent with actin’s role as the main anchoring site for these and other postsynaptic proteins^46^. Our data further indicate that these changes are mediated by drebrin, which binds to F-actin to slow the depolymerization of filaments, the core element of dendritic spines critical for synapse stability and function^47^. Indeed, drebrin-mediated synapse loss is at the center of other pathological disease states marked by spine loss^28,29^, such as Alzheimer’s disease, for which the marked decrease in drebrin expression has become a hallmark^48^.

Chromatin remodeling is a central mechanism for the regulation of gene transcription in disorders involving synaptic plasticity, and we demonstrate that this occurs following heroin SA, leading to changes in acetylation along the drebrin promoter. Specifically, we observed an increase in the transcriptional repressor HDAC2 and a concomitant decrease in acetylation of the drebrin promoter. These data are in line with studies which note that HDAC2-knockout mice have a higher spine density in the hippocampus than wild types and that HDAC2 overexpression decreased spine density^34^. We further showed that HDAC inhibition reduced HDAC2 binding along the drebrin promoter, leading to enhanced gene transcription, consistent with studies in which HDAC inhibition restored drebrin levels in a mouse model of Alzheimer’s^49^. Most importantly, we showed that HDAC inhibition decreased drug-induced reinstatement, providing the first evidence for HDAC2-mediated loss of drebrin in the pathophysiology of opiate abuse. While we chose an inhibitor (MI-192) that blocks the function of class I enzymes, HDAC2 and HDAC3, MI-192 exhibits higher selectivity for HDAC2. Future, our data are in agreement with previous preclinical studies of Alzheimer’s have identified that overexpression of HDAC2, but not HDAC1 or 3, induces dendritic spine abnormalities^50^, suggesting HDAC2 as a predominant contributor to synaptic plasticity in class I HDAC enzymes.

Following exposure to opiates, there is synaptic rewiring within the NAc, including a decrease in dendritic spine density on MSNs^44^^,^^20,42,51^; but see^52,53^, which persists during prolonged abstinence^11,44^. The weakening of dendritic spines, which is observed through immediate changes in immature, thin spines, leads to a persistent decrease in spine density following prolonged withdrawal^11^. We expand on these findings by demonstrating that these alterations are exacerbated by drebrin knockdown and normalized by drebrin overexpression, which in turn govern actin cycling and relapse-like behavior. Further, consistent with the previous literature, which identified changes in glutamatergic tone as a mechanism of dendritic spine retraction^11^, we also note changes in glutamate receptor expression and function. Importantly, the bidirectionality of drebrin expression to mediate both behavioral and neuronal structural plasticity highlights the essential role of drebrin in synaptic integrity associated with the pathophysiology of addiction. Given that the NAc receives inputs from a variety of regions, including the prefrontal cortex, amygdala, and hippocampus, future studies will investigate the input specificity of altered connectivity to these neurons. Recent studies have demonstrated that the D2 MSNs influence silent excitatory synapses and presynaptic neurotransmitter release^10,11^, whereas single-cell drop-sequencing studies indicate that acute morphine treatment engages D1 MSNs^54^, which leads to enhanced morphine reward^9^. Here we find that heroin SA altered drebrin regulation and spine plasticity exclusively in D1 MSNs. Moreover, D1-specific restoration of drebrin levels reversed the effects on dendritic spine density following drug-primed reinstatement, further supporting the role of D1 MSN activation in drug seeking^55^. While our whole-cell findings note changes in dendritic spine changes and glutamatergic tone, our cell-type specific data cannot rule out the possibility that synaptic pruning occurs in D1-MSNs through loss of drebrin, which leads to increased synaptic strengthening on a per spine basis.

These data provide substantial evidence that heroin induces transcriptional repression of drebrin via HDAC2. Furthermore, this opiate-induced repression promotes drug-seeking behaviors and structural remodeling in D1 MSNs, which can be reversed via virus-mediated gene therapy and pharmacological HDAC inhibition. Overall, this study identifies a novel direction, both from a transcriptional and cell-type-specific perspective, for therapeutic targeting strategies in the reversal of opiate addiction.

## Materials and methods

### Subjects

Subjects included male Sprague-Dawley rats (Envigo Laboratories, Indianapolis, IN). For cell-type specific experiments, transgenic Long-Evans *D1-iCre* (LE-Tg(Drd1a-iCre)3Ottc) and *D2-iCre* (LE-Tg(Drd2-iCre)1Ottc) rats were obtained from the National Institute on Drug Abuse (NIDA transgenic rat project; RRRC, Columbia, MO)^43^ and crossed with wild type (WT) Long-Evans (Charles River, Wilmington, MA). A breeding colony was maintained for the production of D1-cre and D2-Cre heterozygous male and female rats. Rats were 250–300 grams (males) or 175–200 grams (females) at the beginning of experimental procedures. The homozygous RiboTag (RT) mice on a C57BL/6J background, expressing Cre-inducible hemagglutinin (HA)-Rpl22 (Jackson Laboratory, Bar Harbor, ME) were crossed with D1-Cre (line FK150) or D2-Cre (line ER44) mouse lines (GENSAT) to generate D1-Cre-RT and D2-Cre-RT mice^56^. Animals were housed at 22–25 °C under a 12-h/12-h reverse light/dark cycle with lights on at 6:00 pm and *ad libitum* access to food and water. Testing took place 7 days per week during the dark portion of the light/dark cycle. All animals were maintained according to the National Institutes of Health guidelines in Association for Assessment and Accreditation of Laboratory Animal Care accredited facilities. All experiments and protocols were approved and conducted in accordance with the Institutional Animal Care and Use Committee (IACUC) of The State University of New York at Buffalo, University of Maryland School of Medicine, and California State University at Bakersfield.

### Self-administration chambers

The experimental chambers have been described in detail elsewhere^32,35,57^. Briefly, 24 standard Med Associates Inc. chambers (St. Albans, VT), each containing two nose-poke holes with infrared monitoring, were used. Two stimulus lights were mounted above each nose-poke hole, with a house light in the center back wall of the test chamber. All chambers are housed in sound-attenuating boxes and controlled through a Med Associates interface.

### Drugs

Solutions of heroin hydrochloride and morphine (generously gifted from the NIDA drug supply program), dissolved in 0.9% sterile saline, were prepared on a weekly basis (0.07 mg mL^−1^, and 10 mg mL^−1^, respectively). Pump durations and injection volumes were adjusted according to the animals’ body weights on a daily basis to ensure delivery of the correct dose of drug for each animal.

### Jugular catheter and patency

Animals were implanted with chronic indwelling jugular catheters, as previously described^32,35^. To preserve patency, catheters were flushed daily with 0.2 mL of heparinized saline (solution 50 IU mL^−1^) containing enrofloxacin (4 mg mL^−1^). Patency testing took place once per week throughout the experiments. Catheter patency was tested by injecting ketamine hydrochloride (5 mg mL^−1^ in 0.5 mL, i.v.) and observing behavioral responses. The loss of the righting reflex and muscle tone were indicators of patency. Only rats with patent catheters were used in data analyses (<10% exclusion).

### Heroin self-administration

For biochemical experiments, one week after jugular catheter surgery, rats were trained to self-administer heroin (0.02 mg kg^−1^ infusion^−1^ [inf]) or saline for 3 h each day for a total of 10 days (Supplementary Fig. 15a). Responses in the active nose-poke resulted in an infusion of drug, followed by a 20-s time-out period using a fixed ratio (FR) 1 schedule of reinforcement, which was increased daily to an FR3 and maintained at this FR for the remainder of the self-administration protocol. Following each self-administration session, catheters were flushed, and the rats were returned to the colony room. Tissue collection was performed 24 h following the last self-administration session. Subjects were rapidly decapitated, and their brains were harvested and sectioned into 1-mm-thick sections from which 1-mm-diameter biopsy punches were taken in regions as previously described^58^.

### Sucrose self-administration

Rats were trained to self-administer sucrose (10%) or water for 3 h each day for a total of 10 days. Responses in the active nose-poke resulted in the delivery of 88.5 μl of a sucrose solution directly in the active hole accompanied by a 5-s cue light, followed by a 20-s time-out period using a fixed ratio (FR) 1 schedule of reinforcement, which was increased daily to FR3 and maintained at this FR for the remainder of the self-administration protocol.

### Repeated morphine exposure

Animals were treated with saline or morphine (10 mg kg^−1^, i.p.) for 10 days. Twenty-four h following their last injection, tissue punches or whole brains were collected for mRNA or protein analysis.

### Locomotor activity

Locomotor activity was recorded via an infrared motion-sensor system (AccuScan Instruments, Columbus, OH) fitted outside plastic cages (40 × 40 × 30 cm). Plastic cages contained a layer of corn cob bedding and were cleaned between each test session. The Fusion activity-monitoring system software monitors infrared beam breaks at a frequency of 0.01 s, and any interruption of beam that was not interrupted during the previous sample was interpreted as an activity score. The Versa Max animal activity-monitoring software monitored the distance the animal traveled in 1 h.

### Viral constructs

The herpes simplex virus (HSV) system allows for expression of proteins exclusively in neuronal cells^59^ that is detectable in vivo for 8 days, with maximal expression occurring on days 3–4^60^. Drebrin and scrambled control constructs were obtained from Addgene (Watertown, MA)^61^ and cloned into a p1005 backbone under the CMV promoter to produce HSV-drebrin and HSV-scrambled. The CRISPER construct was designed by first selecting guide RNAs (gRNAs) against rat drebrin (ENSG00000113758) using the free software at e-CRISP.org. The resulting gRNAs were cloned into an all-in-one mammalian expression vector in the configuration hCMV-Cas9–2A-GFP-WPRE-U3 promoter-gRNA and were tested in vitro for efficiency of drebrin knockdown and specificity. The construct that gave the greatest knockdown (containing the sequence GCCCGCAGCATGGCCGGCGT) was packaged into virus. For cell-type specific behavioral and morphological studies, drebrin was cloned into a cre-dependent transcription cassette (HSV-LS1L-drebrin), which utilizes a lox-stop-lox to confer cre dependence^62,63^. For rat RiboTag studies, recombinant Cre-dependent adeno-associated virus (AAVs) were used in this study. Cre-inducible RiboTag (HA-Rpl22^64^; gifted from Dr. McKnight) in a EF1a-DIO vector was packaged into AAV (serotype 2)^56^. All viral constructs were validated in vivo.

### Stereotaxic surgeries

Rats were counterbalanced on the basis of their behavioral performance (i.e., number of infusions earned during self-administration) and assigned to receive intra-nucleus accumbens (NAc) injections of HSV-drebrin, HSV-GFP, HSV-CRISPR drebrin, HSV-scrambled, or HSV-LS1L-drebrin. The injectors were set at a 10° angle (measurements on the surface of the skull from bregma taken in mm: AP, +1.7; ML, +2.45; DV, −6.7), and the viruses were manually infused at a rate of 0.2 μl min^−1^ for a total of 1.0 μl hemisphere^−1^. The injectors were left in place for an additional 10 min to allow for diffusion. Behavioral and biochemical assays were performed during maximal viral expression (4 days following surgery) with the exception of those with HSV-CRISPR drebrin, which were performed 1 week following maximal expression to allow for optimal protein turnover and degradation (see Supplementary Fig. 4c–e).

### Drug- and sucrose-induced reinstatement

Animals were trained to self-administer heroin (0.02 mg kg^−1^ inf^−1^), saline, sucrose (10%), or water for 10 days (Supplementary Fig. 15b). Following self-administration (day 11), rats (Sprauge Dawley for HSV-drebrin and HSV-CRISPR experiments and D1-/D2-Cre Long Evans for HSV-LS1L-drebrin experiments) were returned to the experimental chambers for a within-session extinction procedure in which heroin or sucrose administration was withheld, as previously described^32,65^. Briefly, rats were exposed to extinction sessions in the presence of the house light and cues that designated drug/sucrose availability during training sessions. The animals were allowed to respond for 8–10 1-h sessions, separated by 5-min intervals during which the house light was extinguished, until their responses fell to less than 30 responses per session. Following extinction, on day 12, rats were counterbalanced and received viral injections (HSV-drebrin, HSV-GFP, HSV-CRISPR drebrin, HSV-scrambled, or HSV-LS1L-drebrin) in the NAc. Following recovery, when the virus is maximally expressed, rats were then injected with 0.25 mg kg^−1^ of heroin (s.c.) or primed with 0.2 mL of sucrose directly in the active hole, returned to the operant chambers, and tested for drug or sucrose reinstatement responding, respectively.

### Cannula implantation and HDAC2 inhibition

For the study of HDAC2 inhibition, rats simultaneously underwent jugular catheter surgery and implantation with a bilateral guide cannula (C235F-2.4; Plastics One, Roanoke, VA), aimed at the NAc shell (all coordinates from bregma at the surface of the skull: AP, +1.8; ML, +1.2; DV, −6.5 mm). Animals were handled daily and sham injected during the recovery period in order to habituate them to the microinjection procedure. Following 10 days of heroin self-administration (0.02 mg kg^−1^ inf^−1^; described above) and extinction (described above in Drug- and sucrose-induced reinstatement; Supplementary Fig. 15c), animals were counterbalanced according to self-administration performance and assinged to receive microinjections of vehicle or HDAC inhibitor, MI192 (5647; Tocris Bio-Techne, Minneapolis, MN; 1 mM dissolved in a mixture of dimethyl sulfoxide and phosphate-buffered saline [PBS; 1:20; vol vol^−1^] and then diluted 1:1,000 in PBS for a final concentration of 1 μM) for a total of 1.0 μL hemisphere^−1^. Microinjections were infused at a rate of 0.5 μL min^−1^ and injectiors were left in place for 10 min to allow for diffusion. Microinjections occurred for 3 days, prior to drug-induced reinstatement. Twenty-four h following the last microinjections, rats were placed in operant chambers and tested for drug reinstatement responding as previously described.

### Dose response

Rats were first trained to self-administer heroin for 5 days as described above (5 days; 2 h day^−1^; 0.02 mg kg^−1^ inf^−1^ of heroin; Supplementary Fig. 15d). On day 6, animals were subsequently trained on a within-session dose-response procedure, as previously described^32^ with slight modifications. Briefly, the 2-h self-administration session was divided into four 30-min sessions, each proceeded by a 2-min time-out period. Rats were exposed to four doses of heroin (0.006, 0.023, 0.045, and 0.02 mg kg^−1^ inf^−1^) for 30 min. The order for the doses tested was pseudorandomized such that the same doses were never tested in the same order during training. We limited each of the four self-administration sessions to 30 min primarily to match the session duration during training (2 h) and to delineate drug intake at multiple doses of drug while minimizing drug side effects (i.e., sedation, stereotypy, etc.), which may be a confounding variable. The dose of heroin per injection was regulated via adjusting the infusion volumes and pump durations. Following each test session, the catheters were flushed and rats were returned to the colony room. Following the 5 days of dose-response training (on day 11), rats received virus infections (HSV-GFP, HSV-drebrin, HSV-scrambled, or HSV-CRISPR drebrin) in the NAc. Following recovery, when the virus is maximally expressed, animals were placed back in the operant chambers and retested on the within-session dose-response procedure described above.

### Dendritic spine analysis

Rats injected with HSV constructs were killed 4 h after heroin-induced reinstatement tests via transcardial perfusion of PBS (0.9% saline [wt vol^−1^]) followed by a 4% formaldehyde (wt vol^−1^) solution. The brains were immersed in fixative overnight and then stored in PBS with 0.01% sodium azide (wt vol^−1^). The brains were sectioned at 100 μm on a Vibratome (VT1000S; Leica Microsystems, Wetzlar, Germany) and blocked in 3% normal donkey serum (wt vol^−1^) with 0.3% Triton X-100 (vol vol^−1^) for 2 h at 4 °C. Sections were then incubated overnight at 22–25 °C in primary antibody (1:1,000 anti-rabbit GFP; Molecular Probes of Thermo Fisher Scientific, Waltham, MA) diluted in PBS with 3% normal donkey serum and 0.3% Tween 20. Tissue sections were rinsed and incubated overnight at 4 °C in anti-rabbit secondary antibody (1:1,000; Jackson ImmunoResearch Laboratories, West Grove, PA). Immunofluorescence was imaged on an LSM 510 Meta confocal microscope (Carl Zeiss, Oberkochen, Germany) with a 63 × oil-immersion lens objective. Images were acquired with a pinhole set at 1 arbitrary unit and a 1,024 × 1,024 frame size. Dendritic length was measured using ImageJ software, and spine numbers were counted. The average number of spines per 10 μm of dendrite was calculated. An average was obtained from 6–10 neurons per rat (*n* = 3–6 for each of four groups). Medium spiny neurons (MSNs) were located in the NAc shell. Only secondary dendrites that were at least 75 μm from the cell body and that were able to be traced back to the cell body were selected for analysis. Spine type analysis was carried out as previously described^32^. Briefly, Neuron Studio (http://research.mssm.edu/cnic/tools-ns.html) was used to analyze dendritic length, width, spine number, and spine head diameter in three dimensions which allowed for classification into major morphological subtypes correlated with spine structure and function. Investigators were blind to the experimental conditions conducted for all confocal acquisitions and analyses of spines.

### Induction of inflammatory pain

After rats received virus infections in the NAc, inflammatory pain was induced by injecting the foot pads of the right hind paws with 0.1 mL complete Freund’s adjuvant (Thermo Fisher, Waltham, MA), which contains 0.05 mg of *Mycobacterium butyricum* dissolved in paraffin oil.

### Place escape/avoidance paradigm

The place escape/avoidance paradigm, a model of the affective component of pain, was measured as previously described^66^. To examine the effects virus-mediated gene expression had on drug-induced escape/avoidance learning behaviors, morphine (10 mg kg^−1^, i.p.) was administered 30 min before the behavioral testing session. Briefly, rats were placed in inverted cages, painted half white and half black. The cages were placed on an elevated metal grid to enable the visualization and stimulation of the plantar surface of the hind paw with a 60-g von Frey hair every 15 s over a 30-min session. When the rat was on the white side of the chamber, the noninflamed (left) paw was mechanically stimulated; while on the black side, the inflamed paw (right) was mechanically stimulated. The location of the rat in the chamber at the time of stimulation was recorded. The percentage of the time spent on the white side of the chamber was calculated every 15 s during each 5-min period and was used as an indication of escape/avoidance learning.

### Mechanical hyperalgesia and tolerance induction

Mechanical hyperalgesia was assessed as previously described^67^ with minor modifications. For this experiment, animals were treated with saline and then cumulative doses of morphine (1.78, 3.2, 5.6, or 10 mg kg^−1^ [i.p.]). Hyperalgesia was measured using calibrated von Frey filaments (1.4–26 g; North Coast Medical, Morgan Hill, CA). The rats were placed in elevated plastic chambers on a mesh wire floor. Von Frey filaments were applied to the medial plantar surface of the hind paw in ascending order until buckling occurred and maintained for approximately 2 s. The mechanical threshold (expressed as percent maximal possible effect [%MPE]) corresponds to the lowest filament force that elicited a behavioral response (paw withdrawal) in at least two of three applications of the filament. The tests were performed in cumulative dosed multiple-cycle procedures, with measurements taken immediately prior to and 20 min after drug administration before the next drug dose was delivered. These cycles continued until an MPE near 100% was reached (26 g) or until a dose caused generalized behavioral suppression. Forces larger than 26 g would physically elevate a non-complete Freund’s adjuvant-treated paw and therefore are not reflective of pain-like behavior. Immediately following the von Frey testing, tolerance was induced in animals over 3 days as previously described^68^ with minor modifications. Briefly, rats were treated twice daily with escalating doses of morphine (20, 40, 60, 80, and 100 mg kg^−1^) over 3 days. Twenty-four h after the last dose, the rats were tested for mechanical hyperalgesia as described above with saline and cumulative doses of morphine (17.8, 32, 56, and 100 mg kg^−1^, [i.p.]).

### Western blot analysis

NAc tissue punches from each rat that self-administered heroin or saline were homogenized in 25 mM Tris (pH 8.0) and 0.25 M sucrose buffer with phosphatase inhibitor cocktails 2 and 3 (Sigma-Aldrich, St. Louis, MO) and protease inhibitors (Roche, Basel, Switzerland). Samples were centrifuged at 21,000 rpm for 10 min to remove debris. Protein concentrations were determined, and 30 μg was loaded onto a 4–15% gradient Tris-sodium dodecyl sulfate (SDS) gels (Bio-Rad Laboratories, Hercules, CA) for SDS-polyacrylamide gel electrophoresis (PAGE) and then transferred to a BioTrace nitrocellulose membrane (Pall Biotech, Show Low, AZ) and blocked in 5% nonfat milk. All membranes were incubated overnight at 4 °C. Primary antibodies were diluted in blocking buffer (Rockland Immunochemicals, Inc., Limerick, PA), including those against drebrin (1:500; Cell Signaling Technologies, Inc., Danvers, MA), HDAC2 (1:1000; Cell Signaling Technologies, Inc.), actin (1:500; Cytoskeleton, Inc., Denver, CO), GluA1 (1:100; Santa Cruz Biotechnology, Dallas, TX), NR2B (1:500; Millipore, Darmstadt, Germany), NR1 (1:500; Millipore), and GAPDH (1:10,000; Cell Signaling Technologies, Inc.). Membranes were incubated for 1 h at room temperature with IRDye secondary antibodies (1:5,000; LI-COR, Inc., Lincoln, NE). The membranes were imaged using the Odyssey infrared imaging system (LI-COR, Inc.) and quantified by densitometry using Image J (National Institute of Health, Bethesda, MD). Full length blots for the main text can be found in Supplementary Fig. 16.

### F/G-actin in vivo assay

The ratio of F actin to G actin in cells was analyzed using an F-actin/G-actin in vivo assay kit (catalog number BK037; Cytoskeleton Inc.) according to the manufacturer’s protocol. Briefly, NAc tissue punches, collected 30-min after the last injection, were lysed w with a motorized pestle homogenizer in a heated (37 °C) solution containing lysis and F-actin stabilization buffer (catalog number LAS01), ATP (catalog number BSA04) and protease inhibitor cocktail (catalog number PIC02). Following a 10 min incubation at 37 °C lysates were centrifuged (350 x g) for 10 min to pellet debris. Supernatants were collected and centrifuged at 100,000 × *g* for 60 min at 37 °C. The supernatants (G-actin) were separated from the pellets (F-actin) and were immediately placed on ice. The pellets (F-actin) were resuspended in F-actin depolymerizing buffer (catalog number FAD02) and incubated on ice for 1 h, pipetting up and down every 15 min. It should be noted differences in resuspension buffers result in different band widths during SDS-PAGE separation. Equal amounts of the samples were loaded in each lane and analyzed by Western blotting with an anti-actin antibody (catalog number AAN01).

### Polyribosome immunoprecipitations and RNA isolation

Polyribosomes were immunoprecipitated as previously described^56^. The NAc of D1-Cre rats and D2-Cre rats, which received bilateral injections of Cre-inducible RiboTag (HA-Rpl22^64^), were dissected under basal conditions for validation purposes (Supplementary Fig. 11) or after heroin self-administration. The NAc of D1-Cre-RT and D2-Cre-RT mice was dissected following repeated i.p. morphine administration. Four 14-gauge NAc punches were pooled from one or four animals, respectively and homogenized by douncing in a supplemented homogenization buffer (50 mM Tris, 100 mM KCl, 12 mM MgCl_2_, 1% NP-40, 1 mM dithiothreitol, 100 ug mL^−1^ cyclohexamide, 1 mg mL^−1^ heparin, 200 U mL^−1^ RNasin, and protein inhibitors). The supernatants (800 μl) were added directly to HA-coupled beads and rotated overnight at 4 °C. The beads were then washed three times for 5 min in a high-salt buffer (50 mM Tris [pH 7.4], 300 mM KCl, 12 mM MgCl_2_, 1% NP-40, 1 mM dithiothreitol, 100 ug mL^−1^ cyclohexaminde) on a magnetic rack. Following the washes, RNA was extracted using the TRK lysis buffer provided in the MicroElute total RNA kit (Omega Bio-tek, Inc., Norcross, GA) according to the manufacturer’s instructions. RNA was quantified by using a NanoDrop spectrophotometer (ND-100; Thermo Fisher Scientific, Hampton, NH). cDNA synthesis and quantitative PCR (qPCR) were carried out as described below.

### RNA isolation and qPCR

NAc tissue punches were collected 1 day after the last heroin administration and immediately stored at −80 °C. RNA was isolated and purified from these samples using Trizol (Invitrogen, Carlsbad, CA) and the MicroElute total RNA kit (Omega Bio-tek, Inc, Norcross, GA) with a DNase step. RNA concentrations were measured on a NanoDrop spectrophotometer, and 500 ng cDNA was then synthesized using an iScript cDNA synthesis kit (Bio-Rad Laboratories, Hercules, CA). mRNA expression changes were measured by RT-PCR with IQ SYBR Green Supermix (Bio-Rad Laboratories, Hercules, CA) and quantified using an iQ5 system (Bio-Rad Laboratories, Hercules, CA). Reactions were run in triplicates using the ΔΔ*C*_*T*_ method as described previously using *Gapdh* as a housekeeping gene. All primer sequences can be found in Table S1.

### Chromatin immunoprecipitation (ChIP)

ChIP was performed for anti-HDAC2, and anti-acetylated H3 as previously described^12,69^ with minor modifications. Briefly, four NAc punches from two rats taken 24 h following the last self-administration session were pooled for each sample. These punches were immediately fixed in 1% formaldehyde for 12 min and then quenched in 2 M glycine for 5 min. Chromatin was sheared using a Biorupter pico 300 (Diagenode Diagnostics, Seraing, Belgium) at 4 °C at a high sonication intensity for 30-s on and 30-s off for 10 min followed by a rest, which was repeated a total of three times. Chromatin fragment sizes of 250–1,000 bp were verified by agarose gel electrophoresis. Magnetic sheep anti-rabbit beads (Invitrogen, Carlsbad, CA) were incubated with anti-HDAC2 (Abcam, Cambridge, MA), anti-acetylated H3 (Millipore, Billerica, MA), and anti-acetylated H4 (Millipore, Billerica, MA) antibodies rotating overnight at 4 °C. After washing, 70 μL of the magnetic bead and antibody slurry was incubated with sheared chromatin for 16 h at 4 °C; 5% of the sheared chromatin was saved as an input control. The samples were washed with lithium chloride and Tris-ethylenediaminetetraacetic acid (EDTA) buffer. Reverse cross-linking took place overnight at 65 °C, and proteins and RNA were removed using proteinase K (Invitrogen, Carlsbad, CA) and RNase (Roche, Basel, Switzerland), respectively. DNA was purified using a DNA purification kit (Qiagen, Hilden, Germany). Immunoglobulin G was used to control for nonspecific binding. qPCR was performed to determine if HDAC2, and acetyl H3 were bound to proximal promoter regions of *Dbn1*. Amplification reactions were run in triplicates with iQ SYBR green (Bio-Rad Laboratories, Hercules, CA), and each sample was normalized to an immunoglobulin G control. Fold changes in expression in animals treated with heroin were calculated relative to that of the saline controls. Primer sequences were tested for efficiency and can be found in Table S2.

### Biotinylation assay

Surface AMPA and NDMA receptors were detected as previously described^65,70^. Briefly, bilateral NAc slices were incubated in artificial cerebral spinal fluid containing 1 mg ml^−1^ NH-S-S biotin for 30 min at 4 °C. Glycine was added to the mixture to quench the reaction, washed three times in lysis buffer (25 mM HEPES, 500 mM NaCl, 2 mM EDTA, 1 mM phenylmethyl sulfonyl fluoride, 20 mM NaF, 0.1% NP-40, protease inhibitor) and homogenized. Cell homogenate was incubated with Neutravidin agarose beads overnight at 4 °C. Following washes, the bound fraction was collected and analyzed by SDS-PAGE as described above.

### Electrophysiological recordings

Whole-cell patch-clamp experiments were performed with a Multiclamp 700 A amplifier and Digdata 1322 A data acquisition system (Molecular Devices, San Jose, CA). Following 10 days of heroin SA, animals were bilaterally injected with HSV-GFP or HSV-drebrin. On day 4, during maximal viral expression, coronal slices (350 μm) containing the NAc were prepared using a Vibratome. The slices were placed in a holding chamber containing artificial cerebrospinal fluid (130 mM NaCl, 26 mM NaHCO_3_, 3 mM KCl, 5 mM MgCl_2_, 1.25 mM NaH_2_PO_4_, 1 mM CaCl_2_, and 10 mM glucose [pH 7.4]; 300 mOsm) oxygenated with 95% O_2_ and 5% CO_2_ and were maintained at room temperature for at least 1 h. For recording of GFP-labeled NAc MSNs, one slice was positioned in a perfusion chamber attached to the fixed stage of an upright microscope (Olympus, Tokyo, Japan) and submerged in continuously flowing oxygenated artificial cerebrospinal fluid. A bipolar stainless-steel stimulating electrode (FHC, Bowdoin, ME) was placed 100 µm rostral to the recording electrode. An S48 stimulator and photoelectric stimulus isolation unit (SIU7) were used to drive the stimulating electrode. The patch electrodes contained an internal solution comprising 130 mM Cs-methanesulfonate, 10 mM CsCl, 4 mM NaCl, 10 mM HEPES, 1 mM MgCl_2_, 5 mM EGTA, 2.2 mM QX-314, 12 mM phosphocreatine, 5 mM ATP, 0.5 mM GTP at pH 7.2–7.3 and 270–280 mOsm. The resistance of the patch electrodes was 3.0 MΩ. Tight seals (>2 GΩ) were obtained by applying negative pressure. The membranes were disrupted with additional suction, and the whole-cell configuration was obtained. The membrane capacitance of all recorded cells was between 40 and 50 pF and the series resistance was <20 MΩ. AMPAR- and NMDAR-mediated evoked excitatory postsynaptic currents (eEPSCs) and their ratios were obtained in the presence of bicuculline (10 µM) at holding potentials of −70 mV and +40 mV, respectively. The NMDAR-mediated eEPSC was calculated 40 ms after the stimulus artifact. The stimulation intensity was set at 0.2 ms and 8 V for the S48 stimulator and at the 10–150 µA range of isolation unit SIU7. Under this stimulation intensity, the AMPAR-mediated eEPSCs from most of the control cells were between 200 and 400 pA. The stimulation intensity setting was kept the same for all eEPSC recordings. Each evoked response was for 3 min with an interstimulus interval of 20 s for all the cells measured. Data analyses were performed with Clampfit software (Axon Instruments of Molecular Devices).

### Statistical analyses

All statistical analyses were performed using GraphPad Prism (GraphPad Software, San Diego, CA). The primary dependent measures were the number of infusions for self-administration (drug and sucrose), the number of total active responses during drug-induced reinstatement, spine density, fold change (mRNA), relative density (protein), and fold change (ChIP). Shapiro-Wilks tests of normality and Bartlett’s tests of homogeneity of variance were conducted to test for normal distributions. In events that a normal distribution could not be assumed, nonparametric tests were used. Performance during self-administration was analyzed using a repeated measures two-factor within-subject analysis of variance (ANOVA), with drug (heroin or saline) as the between-session variable and time (day of testing) as the within-subject variable using Fishers least significant difference (LSD) *post hoc* tests to determine the source of significance. Performance during the within-session dose response was analyzed using a two-factor ANOVA, with virus (HSV-GFP, HSV-drebrin, HSV-scrambled, or HSV-CRISPR-Cas9) as the between-session variable and dose as the within-subject variable, using Fisher’s LSD *post hoc* tests to determine the source of significance. Performance during drug reinstatement and subsequent spine density changes were analyzed using a two-way ANOVA, with virus (HSV-GFP, HSV-drebrin, HSV-scrambled, or HSV-CRISPR-Cas9) and drug treatment (heroin or saline) as the dependent variables; Fisher’s LSD *post hoc* tests to determine the source of significance. Electrophysiological recordings were analyzed using a one-way ANOVA on ranks with Dunn’s *post hoc* analysis. Student’s *t* tests were conducted on measures of locomotor activity, Western blotting data, and mRNA levels followed by Fisher’s LSD *post hoc* tests. Tests were two tailed except where directional hypotheses could be inferred. Significance was set at a *P* value of <0.05, and data are presented as the means ± SEMs.

### Reporting summary

Further information on research design is available in the Nature Research Reporting Summary linked to this article.

## Supplementary information

Supplementary Information

Reporting Summary

## Data Availability

The data that support the findings of this study are available from the corresponding author upon reasonable request.
