# Supplementary material for: A novel role for the actin-binding protein drebrin in regulating opiate addiction
Source: Nat Commun. 2019 Sep 12;10:4140. doi: 10.1038/s41467-019-12122-8 (PMC6742638; doi:10.1038/s41467-019-12122-8)
Supplement: Supplementary file 1 — Supplementary Information [file 41467_2019_12122_MOESM1_ESM.pdf]

A novel role for the actin-binding protein drebrin in regulating opiate addiction

Martin et al.

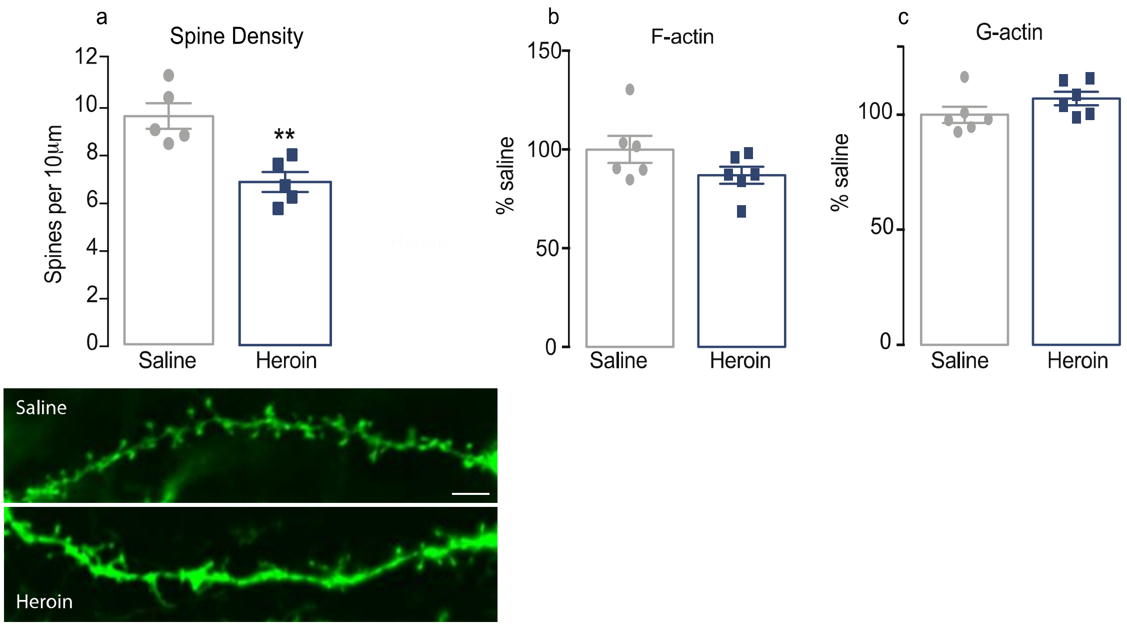

Supplementary Figure 1. Altered actin dynamics in the NAc. (**a**) Dendritic spine density (Student's t-test: $t_8 = 4.364$, $P = 0.002$, n = 5 per group; ×64 magnification; scale bar, 5 μm), (**b**) F-actin, (Student's t-test: $t_{10} = 1.635$, $P > 0.05$, $n = 6$ per group), and (**c**) G-actin (Student's t-test: $t_{10} = 1.542$, $P > 0.05$, $n = 6$ per group) in the NAc following heroin self-administration. Data are expressed as means ± SEMs. $^{**}P < 0.01$.

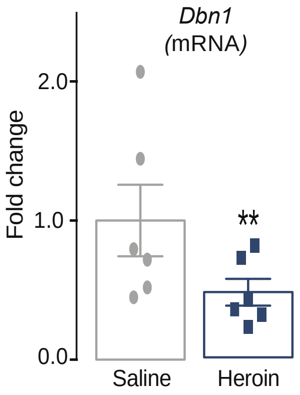

Supplementary Figure 2. Drebrin expression in the NAc. Drebrin mRNA expression in the NAc following heroin self-administration (Student's t-test: t_{10} = 1.93, $P < 0.049$, n = 6 per group). Data are expressed as means ± SEMs. **$P < 0.01$.

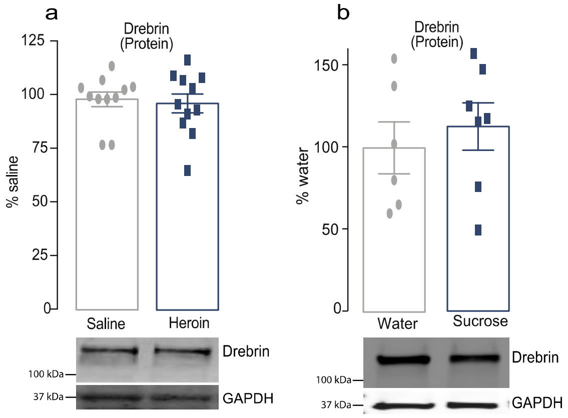

Supplementary Figure 3. Specificity of drebrin expression. (**a**) Drebrin protein expression in the caudate putamen following 10 days of heroin self-administration (Student's t-test: $t_{20} = 0.335$, $P > 0.05$, $n = 11$ per group). (**b**) Drebrin expression in the NAc following sucrose self-administration (Student's t-test: $t_{11} = 0.584$, $P > 0.05$, $n = 6–7$ per group). Data are expressed as means ± SEMs.

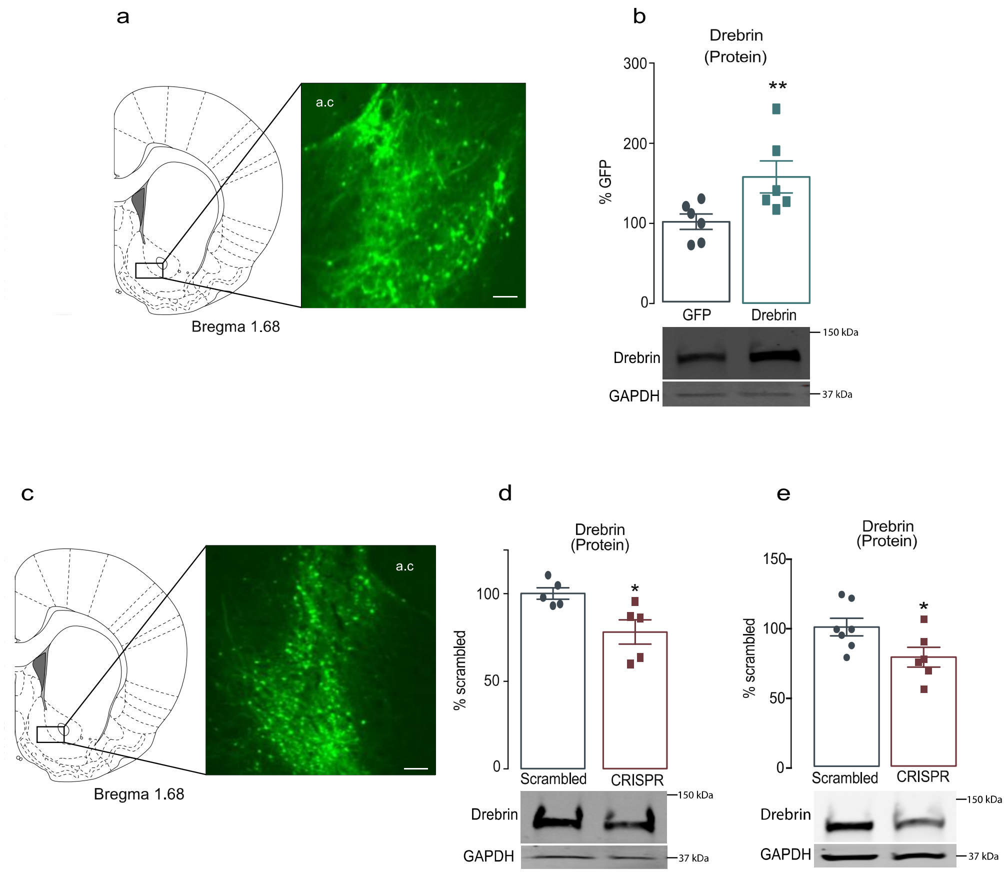

Supplementary Figure 4. Virus-mediated overexpression and knockdown of drebrin in the NAc. Representative picture of GFP expression (×4 magnification; scale bar, 100 μm) (**a**) and drebrin protein level (Student's t-test: t_{10} = 2.587, P < 0.05, n = 6 per group) (**b**) in the NAc following virus-mediated gene transfer. Representative image of CRISPR-Cas9-infected cells expressing GFP in the NAc (×4 magnification; scale bar, 100 μm) (**c**) and drebrin protein levels both in drug-naïve animals (Student's t-test: t_8 = 2.867, P < 0.05, n = 5 per group) (**d**) and in animals that have self-administered heroin (Student's t-test: t_{11} = 3.204, P = 0.042, n = 5–7 per group) (**e**). Data are expressed as means ± SEMs. *P < 0.05, **P < 0.01.

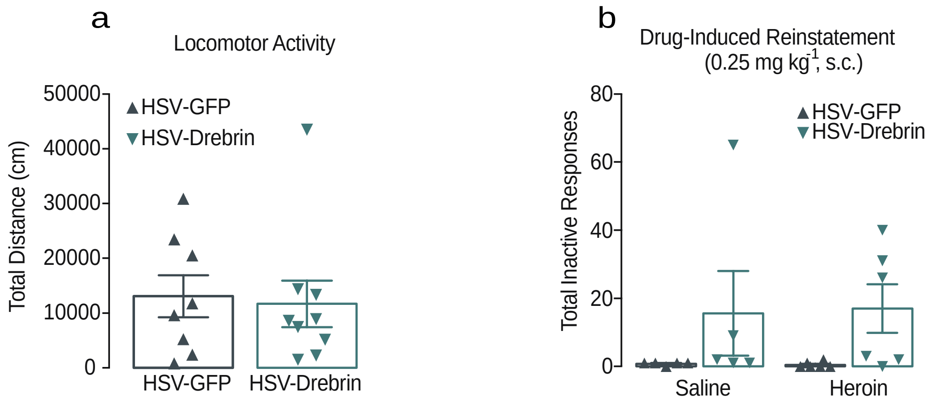

Supplementary Figure 5. Intra-NAc drebrin does not suppress locomotion or operant responding. (**a**) Basal locomotor activity following intra-NAc expression of drebrin (Student's t-test: t_{15} = 0.246, P > 0.05, n = 8–9 per group). (**b**) Total inactive responses during drug-primed reinstatement (two-way ANOVA: drug effect, $F_{1,18}$ = 0.006, P > 0.05; virus effect, $F_{1,18}$ = 5.21, P < 0.05; interaction, $F_{1,18}$ = 0.015, P > 0.05; saline-GFP vs saline-drebrin, P = 0.161; heroin-GFP vs heroin-drebrin, P = 0.09; n = 5–6 per group). Data are expressed as means ± SEMs.

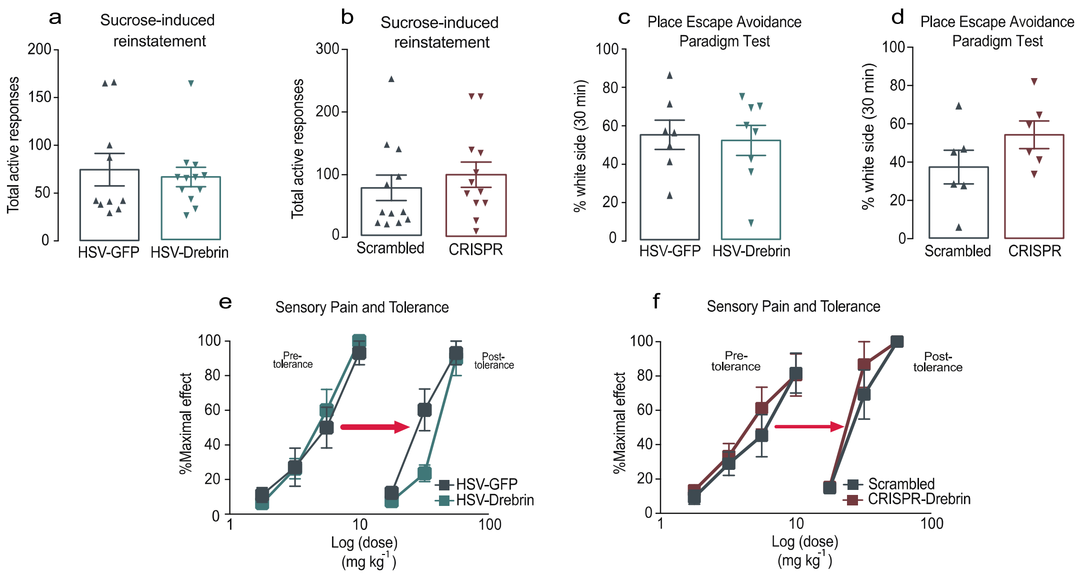

Supplementary Figure 6. Behavioral specificity of intra-NAc drebrin overexpression. Total active responses during sucrose-induced reinstatement after injections of HSV-drebrin or HSV-GFP (Student's t-test: $t_{20} = 0.46$, $P > 0.05$, n = 10–12 per group) (**a**) and HSV-CRISPR-drebrin or HSV-scrambled (Student's t-test: $t_{22} = 0.436$, $P > 0.05$, n = 12 per group) (**b**) in the NAc. Time spent on the white (nonpreferred) side of a chamber during a place escape avoidance paradigm test following virus-mediated expression of drebrin (Student's t-test: $t_{13} = 0.290$, $P > 0.05$, n = 7–8 per group) (**c**) or CRISPR (Student's t-test: $t_{10} = 0.168$, $P > 0.05$, n = 6 per group) (**d**) in the NAc. Percent maximal possible effect of inflammation-induced mechanical nociception and tolerance following intra-NAc expression of HSV-drebrin (two-way ANOVA: dose effect, $F_{6,98} = 43.58$, $P < 0.001$; virus effect, $F_{1,98} = 1.334$, $P > 0.05$; interaction, $F_{6,98} = 1.943$, $P > 0.05$, n = 8 per group) (**e**) or CRISPR (two-way ANOVA: dose effect, $F_{6,70} = 30.62$, $P < 0.001$; virus effect, $F_{1,70} = 1.563$, $P > 0.05$; interaction, $F_{6,70} = 0.373$, $P > 0.05$, n = 6 per group) (**f**) in the NAc. Data are expressed as means ± SEMs.

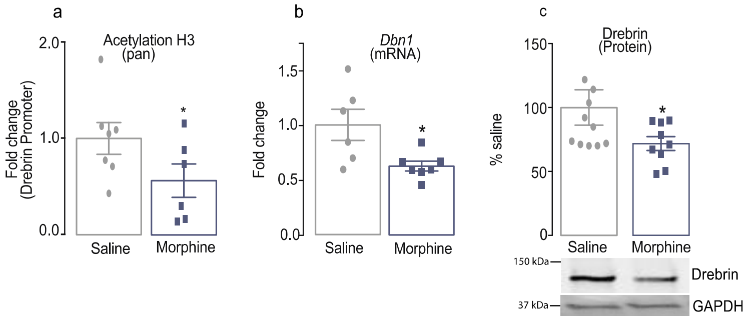

Supplementary Figure 7. Morphine-induced decreases in acetylation and drebrin expression. (**a**) Levels of H3 and H4 (pan) acetylation along the drebrin promoter following morphine exposure (Student's t-test: t_{11} = 1.812, $P < 0.05$, n = 6–7 per group). Drebrin mRNA (Student's t-test: t_{11} = 2.655, P = 0.022, n = 6–7 per group) (**b**) and protein (Student's t-test: t_{18} = 1.752, P = 0.048, n = 9–10 per group) (**c**) expression of levels in the NAc following morphine exposure. Data are expressed as means ± SEMs. *$P < 0.05$

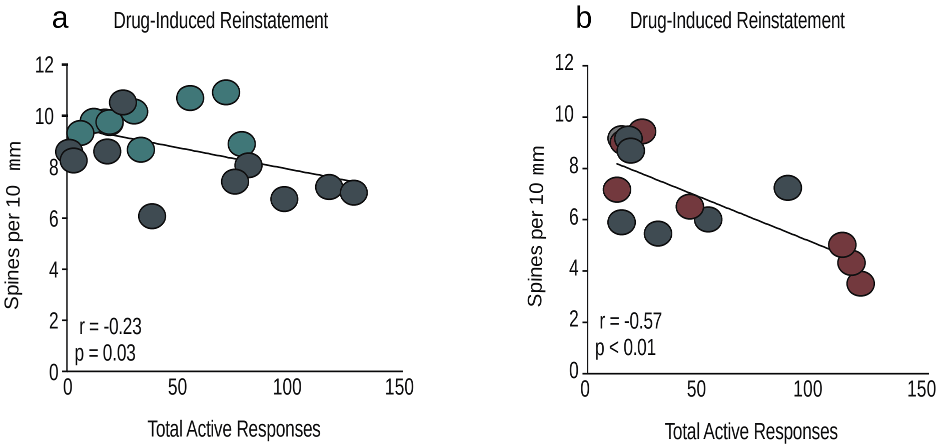

Supplementary Figure 8. Reinstatement activity correlates with morphological plasticity in the NAc. (**a**) Correlation between responding during drug-primed reinstatement and dendritic spine density in the NAc (Pearson's correlation: r = -0.223, $P < 0.05$) following drebrin overexpression. (**b**) Correlation between responding during drug-primed reinstatement and dendritic spine density in the NAc (Pearson's correlation: r = -0.571, $P < 0.01$) following CRISPR-Cas9 expression in the NAc.

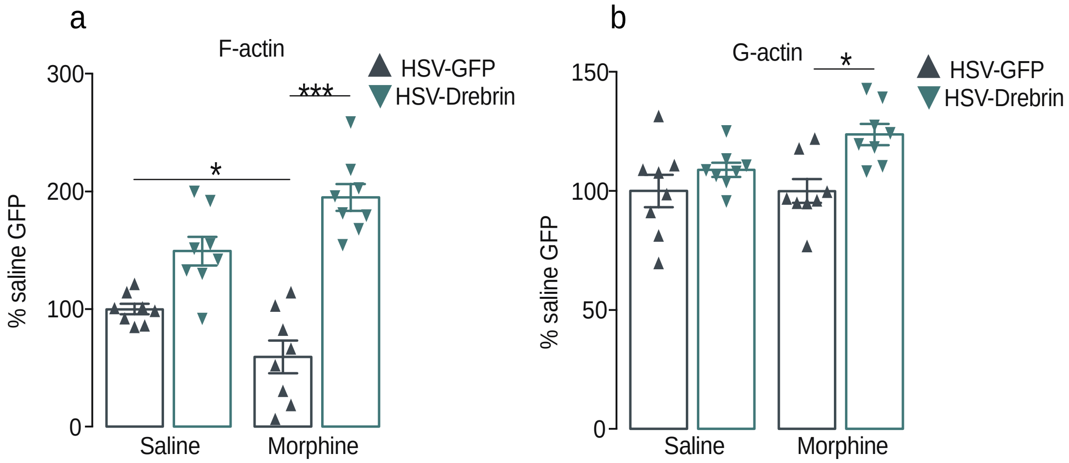

Supplementary Figure 9. Drebrin restores actin dynamics in the NAc. Drebrin restores morphine-induced changes in F-actin pools (two-way ANOVA: drug effect, $F_{1,28} = 0.04$, $P > 0.05$; virus effect, $F_{1,28} = 68.8$, $P < 0.001$; interaction, $F_{1,28} = 14.93$, $P < 0.001$; saline-GFP vs saline-drebrin, $P = 0.004$; saline-GFP vs morphine-GFP, $P = 0.01$; morphine-GFP vs morphine-drebrin, $P < 0.0001$; $n = 8$ per group) (**a**) and G-actin pools (two-way ANOVA: drug effect, $F_{1,28} = 2.16$, $P > 0.05$; virus effect, $F_{1,28} = 10.71$, $P < 0.01$; interaction, $F_{1,28} = 2.21$; saline-GFP vs saline-drebrin, $P = 0.217$; saline-GFP vs morphine-GFP, $P = 0.99$; morphine-GFP vs morphine-drebrin, $P = 0.002$; saline-GFP vs morphine-drebrin $P = 0.002$; $n = 8$ per group) (**b**) following intra-NAc expression of HSV-drebrin. Data are expressed as means ± SEMs. *$P < 0.05$, ***$P < 0.001$.

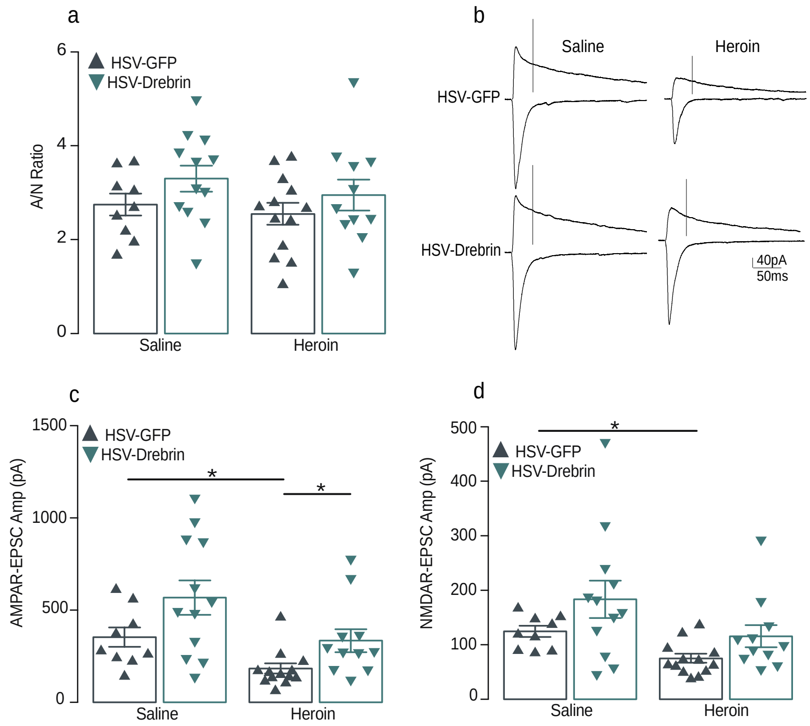

Supplementary Figure 10. Drebrin in the NAc reverses opiate-induced dampening of glutamatergic tone. (**a**) AMPA/NMDA ratios (one-way ANOVA: $F_{4,45} = 3.608$, $P > 0.05$, n = 9–13 cells per group); (**b**) representative traces from each treatment. AMPAR (one-way ANOVA: $F_{1,44} = 15.86$, $P < 0.01$; saline-GFP vs saline-drebrin, $P = 0.33$; saline-GFP vs heroin-GFP, $P = 0.0098$; heroin-GFP vs heroin-drebrin, $P = 0.02$; saline-GFP vs heroin-drebrin, $P = 0.63$; n = 9–13 cells per group) (**c**) and NMDAR (one-way ANOVA: $F_{4,,45} = 13.31$, $P < 0.05$; saline-GFP vs saline-drebrin, $P = 0.57$; saline-GFP vs heroin-GFP, $P = 0.0094$; heroin-GFP vs heroin-drebrin, $P = 0.09$; saline-GFP vs heroin-drebrin, $P = 0.32$, n = 9–13 per group) (**d**) excitatory postsynaptic currents (EPSCs) from MSNs following drebrin overexpression in the NAc. Data are expressed as means ± SEMs. *$P < 0.05$.

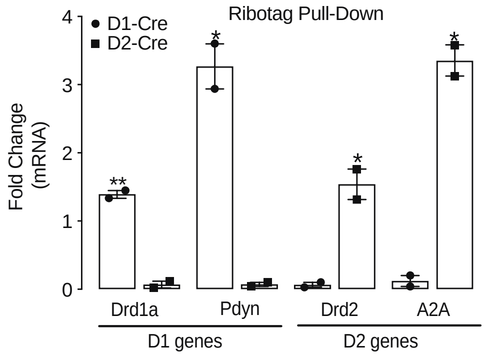

Supplementary Figure 11. Validation of D1- and D2-cre transgenic rats. Cell-type-specific mRNA expression in D1-containing and D2-containing MSNs of Dopamine receptor 1 (Drd1a; Student's t-test: t_2 = 17.50, *P = 0.012, n = 2* per group), Prodynorphin (Pdyn; Student's t-test: t_2 = 9.641, *P = 0.021, n = 2* per group), Dopamine receptor 2 (Drd2; Student's t-test: t_2 = 6.531, *P = 0.022, n = 2* per group) and Adenosine receptor 2a (A2A; Student's t-test: t_2 = 13.40, *P = 0.016, n = 2* per group). Data are expressed as means ± SEMs. **P* < 0.05, ***P* < 0.01

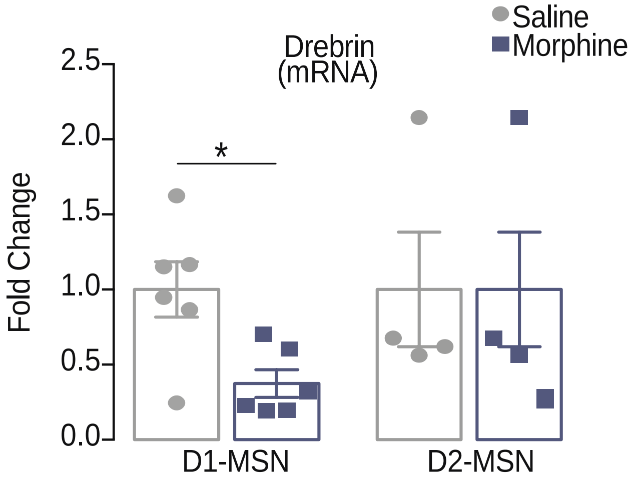

Supplementary Figure 12. Morphine-induced decreases in drebrin expression specifically in D1-MSNs. Cell-type-specific drebrin mRNA expression in D1-containing (Student's t-test: $t_{10} = 3.033$, $P < 0.05$, $n = 6$ per group) and D2-containing (Student's t-test: $t_6 = 0.00$, $P > 0.05$, $n = 4$ per group) MSNs. Data are expressed as means ± SEMs. *$P < 0.05$.

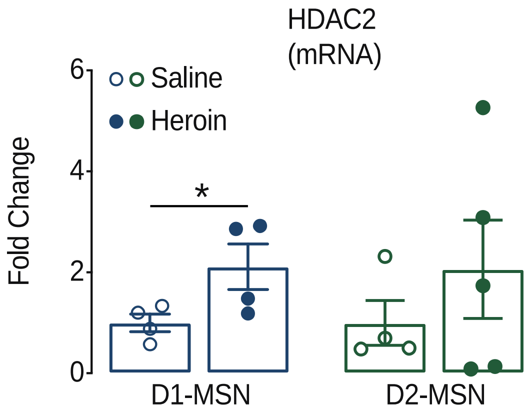

Supplementary Figure. 13. Heroin-induced increase in HDAC2 expression specifically in D1-MSNs. Cell-type-specific HDAC2 mRNA expression in D1-containing (Student's t-test: $t_6 = 2.298$, $P < 0.05$, $n = 4$ per group) and D2-containing (Student's t-test: $t_7 = 0.9071$, $P > 0.05$, $n = 4–5$ per group) MSNs. Data are expressed as means ± SEMs. *$P < 0.05$.

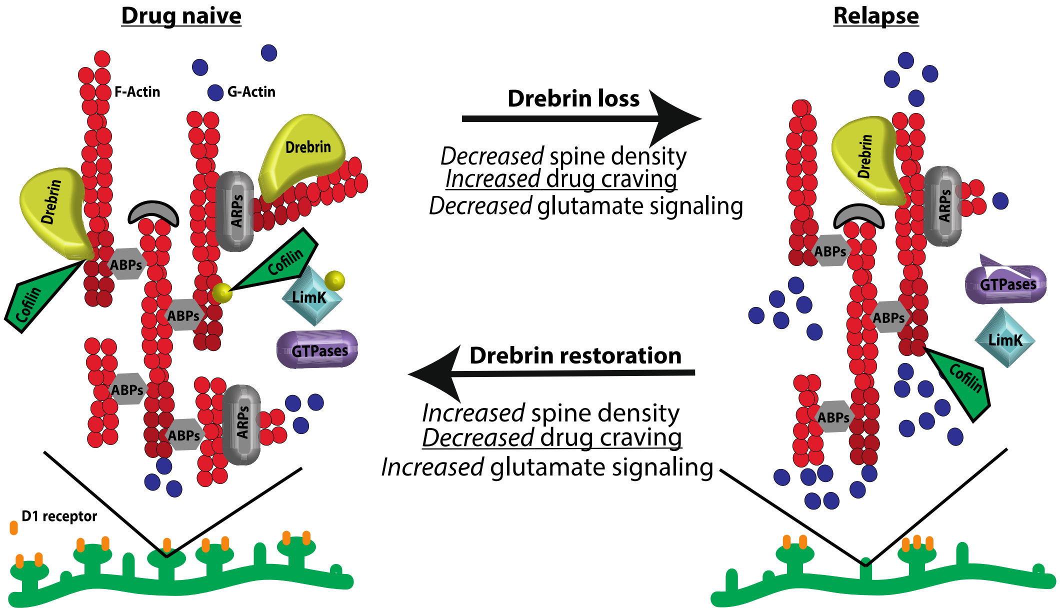

Supplementary Figure 14. Schematic of drebrin signaling in the NAc to regulate opiate-induced plasticity.

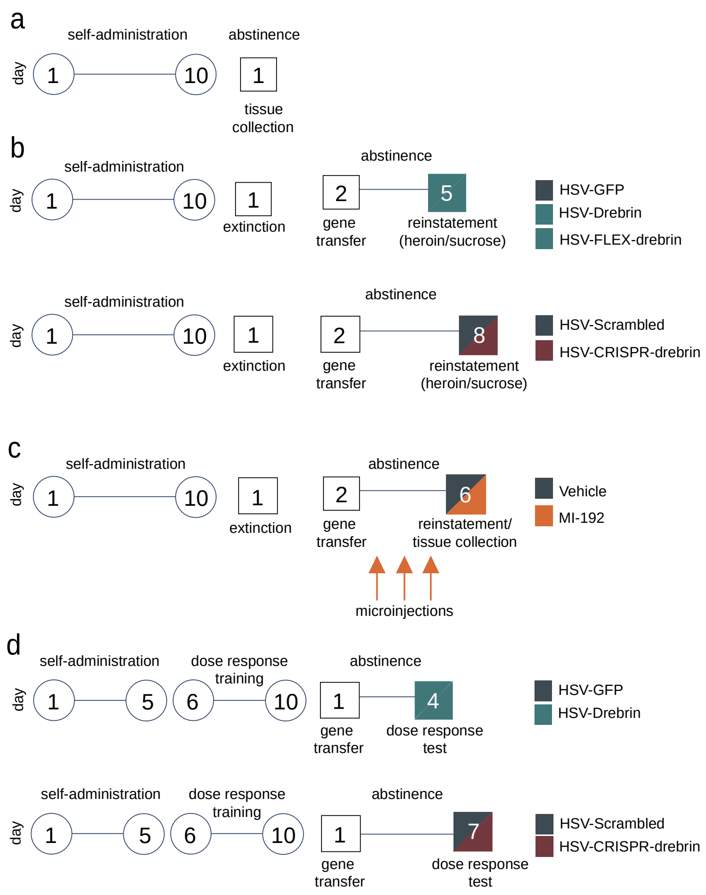

Supplementary Figure 15. Timeline of behavioral experiments. (**a**) Self-administration paradigm for biochemical assays. (**b**) Behavioral paradigm for drug- and sucrose-primed reinstatement. (**c**) Experimental timeline for HDAC2 inhibition studies. (**d**) Timeline for dose-response training and testing.

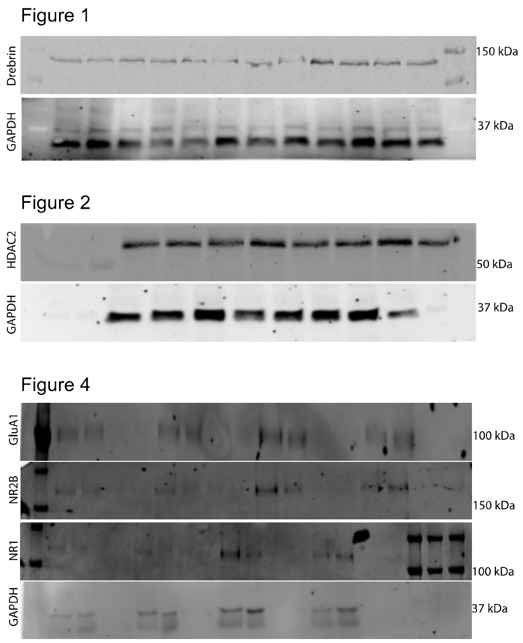

Supplementary Figure 16. Full length blots of main text figures.

Supplementary Table 1: Primer list for mRNA quantification

Gene symbol	Gene name	NCBI Accession No.	Primer sequence, 5'-3'
Dbn1	Drebrin	NM_031024.1	F:GTGACGCACCCTCGCCCTTC
			R:TCCTCCGGTGGCTGTCCAGG
Drd1a	Dopamine receptor D1	NM_012546	F:CCTTCATTCCAGTGCAGCTAA
			R:AGCCAAACCACACAAATACATC
Pdyn	Prodynorphin	NM_019374.3	F:CCATCAACCCCCTGATTTGC
			R:TTGGTCAGTTCCGTGTAGCC
Drd2	Dopamine receptor D2	NM_012547	F:CTCAGGAGCTGGAAATGGAG
			R:AGAGGACTGGTGGGATGTTG
A2A	Adenosine A2A receptor	NM_001357942.1	F: ATTCCACTCCGGTACAATGG
			R: AGTTGTTCCAGCCCAGCAT
Dbn1	Drebrin	NM_001177371.1	F:TGAAGAAGCCAAGAGGAG GTT
			R: ACTCCCGTGGGTTATCAGG
Gapdh	Glyceraldehyde-3-Phosphate dehydrogenase	NM_017008.4	F: AACGACCCCTTCATTGAC
			R: TCCACGACATACTCAGCA

Supplementary Table 2: Chromatin immunoprecipitation primer sequences on the drebrin promoter (Ensembl Accession No. ENSRNOT000000 19569)

Gene name	Binding site on drebrin promoter	Primer sequence, 5′–3′
Histone deacetylase 2	AP-1	F: GGGTCTTGTGAAGTGTAGAGTTT
		R: GCAGGCTCCTGTTCTGATTT
Histone deacetylase 2	SBE	F: TGATGGTGGTAGACTAGGAAGG
		R: GGTCTGTTCCACCCTACATTAAA
Pan H3 acetylation	CBP	F: CCCATCTGATCATCTCTAT
		R: TAGAGATGACATCAGATGGG
