# Supplementary material for: A novel role for the actin-binding protein drebrin in regulating opiate addiction
Source: Nat Commun. 2019 Sep 12;10:4140. doi: 10.1038/s41467-019-12122-8 (PMC6742638; doi:10.1038/s41467-019-12122-8)
Supplement: Supplementary file 2 — Reporting Summary [file 41467_2019_12122_MOESM2_ESM.pdf]

nature research

Corresponding author(s): David Dietz, PhD

# Reporting Summary

Nature Research wishes to improve the reproducibility of the work that we publish. This form provides structure for consistency and transparency in reporting. For further information on Nature Research policies, see Authors & Referees and the Editorial Policy Checklist.

## Statistical parameters

When statistical analyses are reported, confirm that the following items are present in the relevant location (e.g. figure legend, table legend, main text, or Methods section).

| n/a | Confirmed | |
|---|---|---|
| ☐ | ☒ | The exact sample size ($n$) for each experimental group/condition, given as a discrete number and unit of measurement |
| ☒ | ☐ | An indication of whether measurements were taken from distinct samples or whether the same sample was measured repeatedly |
| ☐ | ☒ | The statistical test(s) used AND whether they are one- or two-sided<br>*Only common tests should be described solely by name; describe more complex techniques in the Methods section.* |
| ☐ | ☒ | A description of all covariates tested |
| ☐ | ☒ | A description of any assumptions or corrections, such as tests of normality and adjustment for multiple comparisons |
| ☐ | ☒ | A full description of the statistics including central tendency (e.g. means) or other basic estimates (e.g. regression coefficient) AND variation (e.g. standard deviation) or associated estimates of uncertainty (e.g. confidence intervals) |
| ☐ | ☒ | For null hypothesis testing, the test statistic (e.g. $F$, $t$, $r$) with confidence intervals, effect sizes, degrees of freedom and $P$ value noted<br>*Give P values as exact values whenever suitable.* |
| ☒ | ☐ | For Bayesian analysis, information on the choice of priors and Markov chain Monte Carlo settings |
| ☐ | ☒ | For hierarchical and complex designs, identification of the appropriate level for tests and full reporting of outcomes |
| ☐ | ☒ | Estimates of effect sizes (e.g. Cohen's $d$, Pearson's $r$), indicating how they were calculated |
| ☐ | ☒ | Clearly defined error bars<br>*State explicitly what error bars represent (e.g. SD, SE, CI)* |

*Our web collection on statistics for biologists may be useful.*

## Software and code

Policy information about availability of computer code

| | |
|---|---|
| Data collection | Data collected using: Med-PC, ImageJ, VersaMax, Molecular Devices |
| Data analysis | GraphPad Prism version 7; Clampfit |

For manuscripts utilizing custom algorithms or software that are central to the research but not yet described in published literature, software must be made available to editors/reviewers upon request. We strongly encourage code deposition in a community repository (e.g. GitHub). See the Nature Research guidelines for submitting code & software for further information.

## Data

Policy information about availability of data

All manuscripts must include a data availability statement. This statement should provide the following information, where applicable:

- Accession codes, unique identifiers, or web links for publicly available datasets
- A list of figures that have associated raw data
- A description of any restrictions on data availability

All data are available in the manuscript or the Supplementary materials.

# Field-specific reporting

Please select the best fit for your research. If you are not sure, read the appropriate sections before making your selection.

☒ Life sciences ☐ Behavioural & social sciences ☐ Ecological, evolutionary & environmental sciences

For a reference copy of the document with all sections, see nature.com/authors/policies/ReportingSummary-flat.pdf

# Life sciences study design

All studies must disclose on these points even when the disclosure is negative.

| | |
|---|---|
| Sample size | No statistical methods were used to pre-determine samples size, however out sample size are similar or greater to those previously reported, including our own work (Dietz et al., 2012; Gancarz et al., 2015) |
| Data exclusions | For self-administration, any animals that failed patency testing or failed to acquire self-administration (<10 infusions by day 5) were excluded from analysis. GraphPad Prism Version 7 outliers test, built-in as part of the package |
| Replication | We have included both biological and technical replicates in our biochemical work. Behavioral studies were run in multiple cohorts. |
| Randomization | Samples were randomly assigned to experimental groups unless otherwise noted in manuscript. To ensure proper rigor, and that responding for infusions is not a confounding factor in analysis, experimental groups were counterbalanced for responding prior to manipulations (i.e. viral-mediated gene transfer) |
| Blinding | Investigators were blinded during data collection and analysis |

# Reporting for specific materials, systems and methods

## Materials & experimental systems

| n/a | Involved in the study |
|---|---|
| ☒ | ☐ Unique biological materials |
| ☐ | ☒ Antibodies |
| ☒ | ☐ Eukaryotic cell lines |
| ☒ | ☐ Palaeontology |
| ☐ | ☒ Animals and other organisms |
| ☒ | ☐ Human research participants |

## Methods

| n/a | Involved in the study |
|---|---|
| ☒ | ☐ ChIP-seq |
| ☒ | ☐ Flow cytometry |
| ☒ | ☐ MRI-based neuroimaging |

## Antibodies

| | |
|---|---|
| Antibodies used | GFP; Cy3; Drebrin; HDAC2; Acetyl H3; Actin; GluA1; NR2B; NR1; GAPDH |
| Validation | GFP (Gancarz et al., 2015); Cy3 (Gancarz et al., 2015); Drebrin (Zhou et al., 2015); HDAC2 (WB: Laument et al., 2015; ChIP: Montgomery et al., 2007); Acetyl H3 (Renthal et al., 2009); Actin (Manufactures instruction, Peykov et al., 2015); GluA1 (Yuen et al., 2012); NR2B (Yuen et al., 2012); NR1 (Yuen et al., 2012); GAPDH (Scobie et al., 2014). |

## Animals and other organisms

Policy information about studies involving animals; ARRIVE guidelines recommended for reporting animal research

| | |
|---|---|
| Laboratory animals | Male Sprague Dawley (250-300g); Male and Female Long Evans (D1-Cre, D2-Cre; 175-200, 250-300g); Homozygous RiboTag (RT) mice on a C57BL/6J background (20-30g) |
| Wild animals | *Provide details on animals observed in or captured in the field; report species, sex and age where possible. Describe how animals were caught and transported and what happened to captive animals after the study (if killed, explain why and describe method; if released, say where and when) OR state that the study did not involve wild animals.* |
| Field-collected samples | *For laboratory work with field-collected samples, describe all relevant parameters such as housing, maintenance, temperature, photoperiod and end-of-experiment protocol OR state that the study did not involve samples collected from the field.* |
